# Stereochemical Control in the Matteson Reaction

**DOI:** 10.1002/chem.71365

**Published:** 2026-07-24

**Authors:** Hae Mo Lee, Christoph Hirschhäuser

**Affiliations:** ^1^ Faculty of Chemistry Universität Duisburg‐Essen Essen Germany

**Keywords:** boronate homologation, matteson homologation, stereodivergent synthesis

## Abstract

The Matteson homologation is a highly versatile method for the stereoselective insertion of carbenoids into boronic esters. Through iterative application of the process, complex chiral molecules can be synthesized. The stereoselectivity of the reaction is determined by a chiral diol incorporated in the boronic ester. Upon iterative application, this results in an efficient use of chiral pool starting materials. With that, however, comes limited stereochemical variability. This review delves into boronate homologations that can address these particular limitations and highlights unsolved problems in the field.

## Graphical abstract

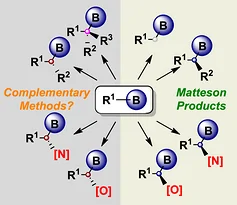

## 1 Boronic Ester Homologations

Iterative syntheses, in which chains of atoms are grown in a controlled, stepwise manner, are ubiquitous in nature [1, 2, 3, 4]. That way great structural diversity can be achieved with only a small number of different transformations. In nature, as in modern synthesis, these processes often rely on enol chemistry, that is, C_2_‐building blocks that combine an adjacent acceptor and donor [5, 6, 7, 8, 9]. A C_1_‐based process would promise even more structural diversity. Therefore, both donor and acceptor functionality must be located on the same atom. Equivalent carbenoids, such as LiCH_2_X, are unstable. However, their reactivity can be tempered by boron‐containing moieties, as shown in Scheme 1.

**SCHEME 1:**
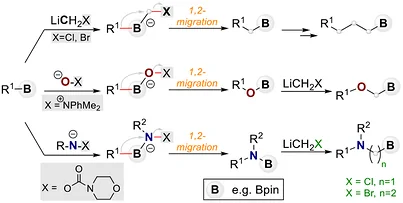
Insertion of carbon, oxygen, and nitrogen into C─B‐bonds.

The electrophilic nature of boron allows for the formation of ate complexes, which stabilizes the carbenoid and activates the nucleophilic C─B bond. This promotes 1,2‐migration of R^1^ resulting in a homologation reaction. Importantly, decomposition of LiCH_2_X usually occurs faster than 1,2‐migration. That way reaction of LiCH_2_X with the 1,2‐migration product and thus further homologation is avoided. This allows for controlled iterative application. The classic oxidation of boranes and boronic esters with H_2_O_2_/NaOH relies on the same principle, but only recently Dong and coworkers found a way to incorporate this process in an iterative sequence. Based on their work even O_1_ [10] and N_1_ [11, 12] building blocks can be inserted nowadays in an iterative synthetic sequence. A key advantage of boronate homologations emerges upon insertion substituted carbon atoms. This way the configuration of each newly introduced stereocenter can be controlled. Chiral auxiliaries can be particularly efficient in this type of chemistry, as they are automatically recycled for each iteration. However, as each stereocenter is directed by the same chiral auxiliary, specialized stereodivergent strategies are required to retain synthetic flexibility. To set the stage, the first part of this review provides an overview of boronate homologations and their fundamental means of exercising stereochemical control. Building on this foundation, the second part of this review will then provide a detailed overview of the methods developed to achieve such stereodivergence in substrate‐controlled reactions.

### 1.1 Substrate‐Controlled Stereoselective Boronate Homologations

In 1963, Matteson and coworkers first described the homologation of boronic esters [13]. The use of chiral diols and carbenoids with two leaving groups (LiCHX_2_) renders the Matteson homologation (MH) stereoselective. Reaction of a chiral boronic ester of type **1** with a carbenoid such as LiCHCl_2_ at a low temperature generates an ate complex of type **2** (Scheme 2) [14, 15, 16, 17, 18, 19, 20, 21, 22, 23, 24].

**SCHEME 2:**
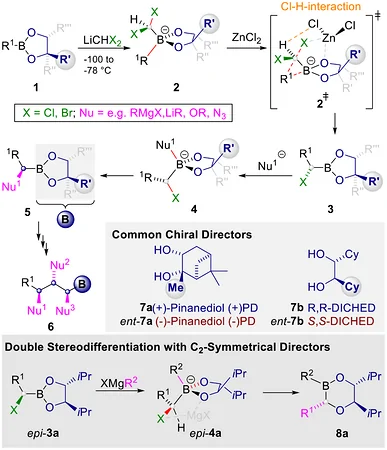
The Matteson homologation (MH).

Subsequent 1,2‐migration occurs diastereoselectively to yield α‐halo boronic esters of type **3**. The addition of ZnCl_2_ is pivotal for obtaining good diastereoselectivities as its coordination to one of the boronate oxygens, as well as one of the diastereotopic chloride atoms leads to the bicyclic transition state **2^‡^**. The highlighted H‐Cl interaction stabilizes the transition state **2^‡^**, which leads to the Matteson product **3**, while a repulsive X‐Cl interaction destabilizes the transition state (not shown) leading to the undesired epimer (*epi*‐**3**) [25, 26]. The resulting halides of type **3** react under inversion to α‐chiral boronic esters of type **5** with a wide variety of carbon nucleophiles, such as Grignard reagents or enolates [18, 27, 28, 29, 30, 31, 32, 33]. Oxygen‐ [27, 34, 35] and nitrogen‐ nucleophiles [27, 36, 37, 38] can be used to introduce masked alcohol and amine substituents. Through iterative application of this process complex chiral molecules (**6**) can be prepared. Two chiral auxiliaries, both enantiomers of which are available, are commonly used. Pinanediol is available from pinene by Upjohn bishydroxylation [39] and usually requires recrystallization as neither enantiomeric form of naturally occurring pinene is enantiomerically pure. The resulting pinanediol esters, however, are of high thermodynamic stability. The C_2_‐symmetrical diol (S,S)‐DICHED (1,2‐**DIC**yclo**H**exyl**E**thane 1,2‐**D**iol) is slightly more reactive and is able to deliver extremely high diastereoselectivity. This was attributed by Matteson to a second stereodifferentiating step, depicted in Scheme 2 [40]. Substitution of **3** usually occurs via an ate complex of type **4**. With C_2_‐symmetrical chrial directors the undesired epimer of α‐halide epi‐3 reacts with Grignard reagents via ate complexes of type *epi*‐**4**. These undergoe a ring‐expanding 1,2‐migration involving one of the boronate oxygen atoms. The resulting borinates (**8a**) usually oxidize and decompose upon aqueous workup. This raises the *de* of the desired homologation product of type (**5**).

A quick heuristic for predicting the correct stereochemical outcome of a Matteson sequence focuses of the moieties highlighted in Scheme 2 (i.e., the CH_3_‐group in pinanediol and any one of the cyclohexyl groups in DICHED). For the depiction of **5** a conformation is chosen, in which the highlighted group on the boronic ester is drawn in proximity to the newly introduced nucleophile. In this configuration both moieties occupy the same hemisphere (i.e., either both point toward or away from the observer). In this review the configuration at newly introduced carbon atoms is color‐coded that is, in a standard reaction a “red chiral director” makes a “red stereocenter,” and a “blue chiral director” makes a “blue one.” Numerous applications of Matteson reactions in organic synthesis have been reported and Figure 1 only shows a small selection.

**FIGURE 1:**
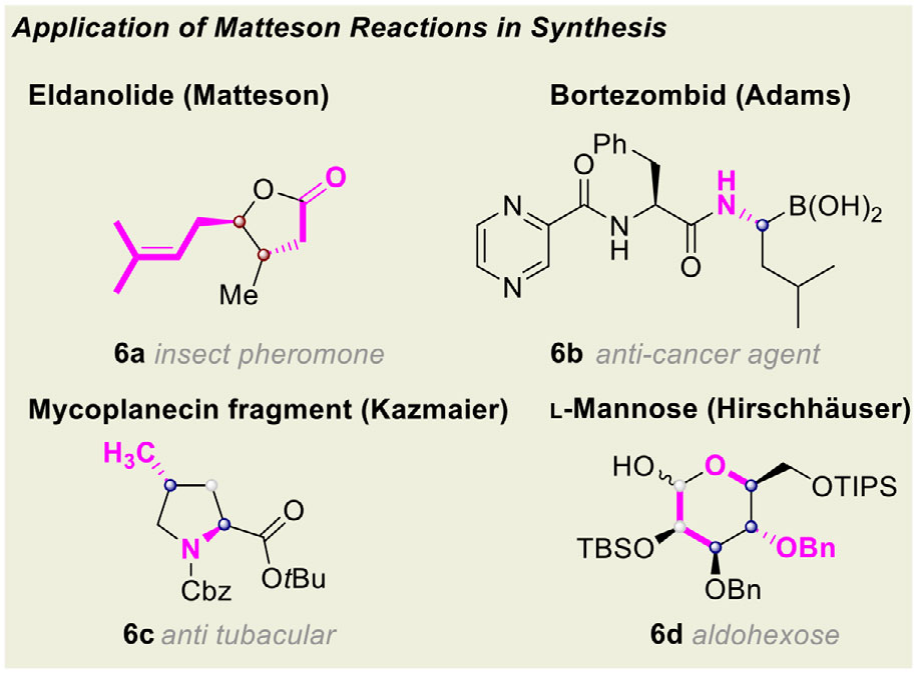
Sample applications of the Matteson reaction in synthesis.

Eldanolide (**6a**), an insect pheromone, was one of the first natural products synthesized by Matteson to demonstrate the synthetic utility of his method [27]. Aminoboronic acids, such as bortezomib (**6b**) are of pharmaceutical interest and can be obtained via substitution of MH products (**3**) with LiHMDS [41]. Sodiumazide is another commonly used N‐nucleophile, which was employed by Kazmaier in the synthesis of mycoplanecin A to prepare proline derivative **6c** [42]. Alkoxides, such as LiOBn, can be used to introduce masked hydroxyl groups. Therefore, sugars appear to be a straight forward target. However, Matteson´s 1987 attempts stopped at ribose due to unforeseen synthetic difficulties [35]. After identifying the formation of 6‐membered intramolecular O‐B‐complexes as a key problem in these heteroatom rich systems, we were able to lay out a strategy for the synthesis of all aldohexoes, such as L‐mannose (**6d**) [43]. More recently a substrate controlled homologation strategy emerged, based on resolution of configurationally labile carbenoids.

During their synthesis of tatanan A [44], the Aggarwal group employed the chiral, but configurationally unstable carbenoid **9** (Scheme 3). It reacted with an α‐chiral pinacol boronic ester of type **10** to the homologation product *S*‐**11** with 78% yield and a *de* of 54%. The selectivity was attributed to the faster formation of ate complex *S*‐**12** over its epimer *R*‐**12**. Subsequent substitution of the OTIB group with an aryl‐Grignard reagent, proceeded in a manner similar to the second step of a Matteson sequence. In it OTIB served as the leaving group. This substitution increased the *de* significantly to 90% delivering *S‐* **13a** in 65% yield (most likely due to kinetic resolution). By using the aryl substituted carbenoid **10** directly, this substitution step and the concomitant inversion can be avoided. This led to the epimeric product *R*‐**13b**. These results clearly show that rate differences in the formation of diastereomeric ate complexes can be exploited to generate stereoselective reactions. This raised the question, whether chiral auxiliaries on the boronic ester could be exploited to generate a more general method [14]. Moreover, could a Curtin‐Hammet equilibrium be exploited to convert both enantiomers of the configurationally unstable carbenoid in an enantioconvergent fashion? As can be seen in Scheme 4, Dong and coworkers provided some synthetically highly useful answers to these questions [45].

**SCHEME 3:**
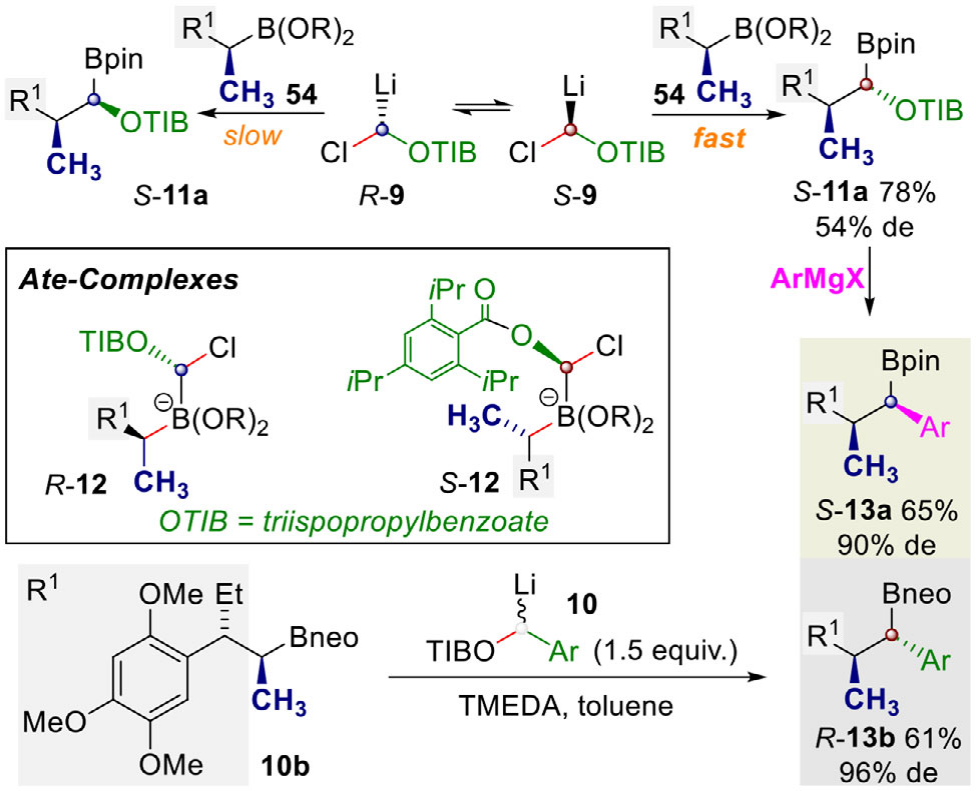
Enantioconvergent reaction of configurationally labile carbenoids allows for diastereoselective homologation.

**SCHEME 4:**
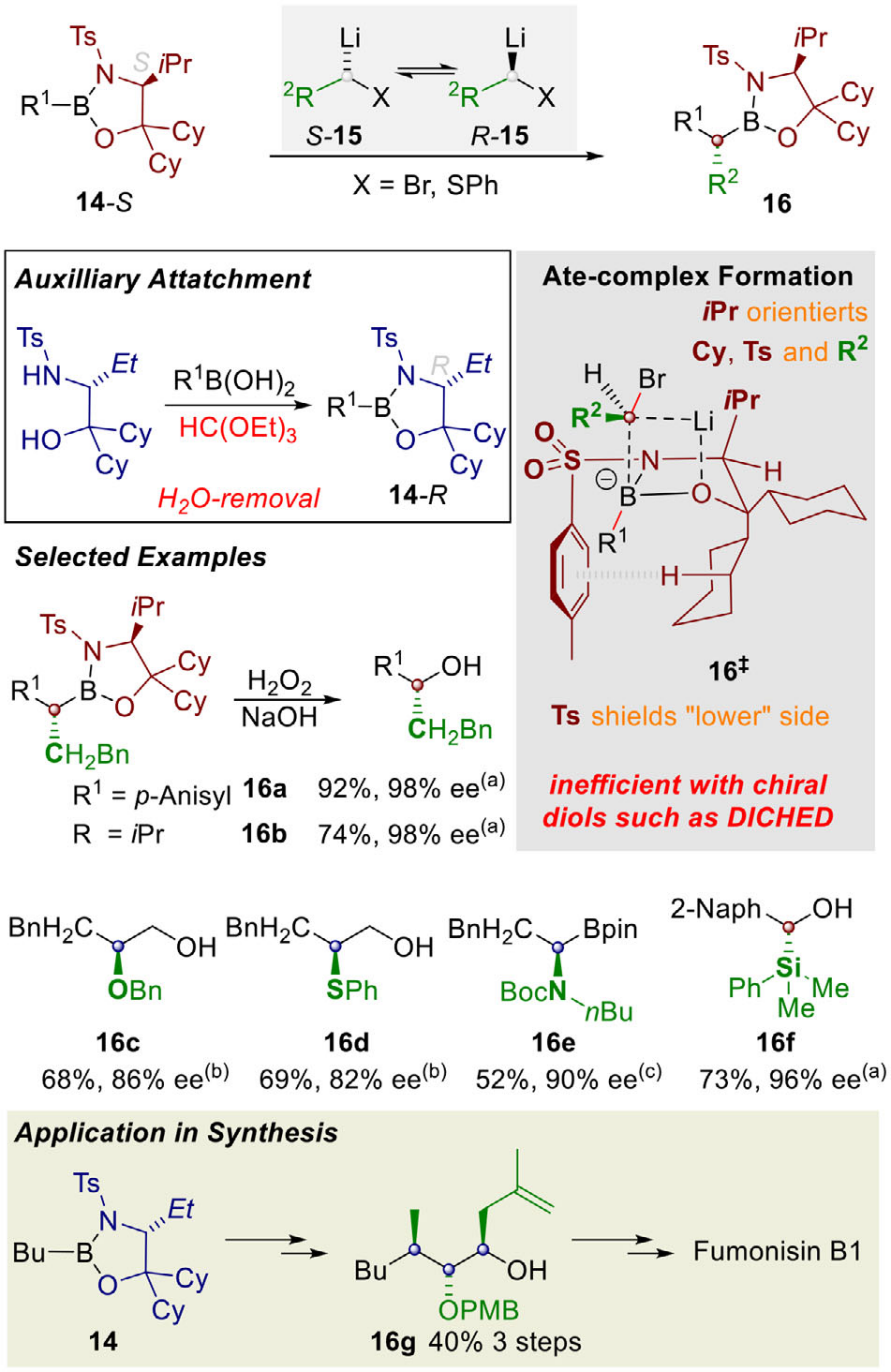
Dong´s enantioconvergent homologation with amino acid derived chiral directors. For isolation insertion of **15** was followed by either (a) oxidation, (b) homologation and oxidation, (c) transesterification.

While the classic Matteson directors (DICHED and pinanediol) proved themselves unsuitable for this approach, chiral oxazaborolidines of type **14** did not. They allowed for the enantioconvergent insertion of racemic carbenoids of type **15** with good diastereoselectivity. As can be seen in **16^‡^**, the N‐tosyl group is of key importance. It exercises its steric influence much closer to the boron atom than the substituents on a chiral glycol such as DICHED. Steric repulsion by the isopropyl moiety orientates the tosyl group toward one side of the oxazaborolidine. This conformation is further stabilized by a π–H interaction, thus blocking an approach to the boron atom from the “lower” side. Consequently, attack of the carbenoid has to proceed from the “top” face. To minimize 1,2‐interactions between the isopropyl‐ and the *syn*‐clinal cyclohexyl group, the oxazaborolidine is distorted. As O‐Li coordination limits the angle of attack for the carbenoid, R^2^ has to point away from the oxazaborolidine to minimize repulsive interactions. This allowed Dong and coworkers to develop a method with an impressive substrate scope. Both aryl and alkyl oxazaborolidines can be homologated with excellent yields and enantioselectivities (**16a** and **16b**). Furthermore, halogen substituents (even in ß‐position) and other C‐H acidic protons are tolerated. The protocol also allows for implementation of various synthetically useful heteroatoms, such as benzylether **16c**, thiophenolether **16d** and Boc‐amine **16e**. Especially the introduction of carbamate protected amines is remarkable, as this requires additional functional group interconversions after introduction of N‐nucleophiles by MH [18, 46, 47, 48]. Silanes such as **16f** can also be made after MH, but have received little attention after Matteson´s initial reports [49, 50]. However, their synthetic use should not be underestimated, as the can serve as masked hydroxyl groups that can be liberated by Tamao–Fleming oxidation [51, 52, 53, 54]. Beyond this Dong's auxiliary can be removed easily by hydrolyzation [45, 55]. This enables a flexible change of the chiral auxiliary (e.g., to **14**‐*R*). That being said, this lower stability toward moisture also makes oxazaborolidines much more sensitive than boronic esters [45, 56]. Nevertheless, by avoiding extensive purification after each individual step, Dong and coworkers managed to perform three consecutive homologations to **16 g**, which was obtained in 40% overall yield and respresents a key intermediate in the synthesis of fumonisin B1 [57].

### 1.2 Reagent Controlled Stereoselective Boronate Homologations

Substrate controlled homologation reactions face an inherent limitation in iterative application, as each newly introduced stereocenter is directed in the same manner. By moving the chiral information away from the substrate to the carbenoid this limitation can be avoided. As depicted in Scheme 5 chiral carbenoids of type **18** can react with boronic esters to form ate complexes of type **19** directly. As rearrangement to ate complexes of type **20** is stereospecific, Blakemore coined the term StReCH (**St**ereoselective **Re**agent **C**ontrolled **H**omologation) [58]. This way the stereoselctive step is shifted to the preparation of the carbenoid or its precursor. Blakemore generated chiral carbenoids of type **18a** from chiral sulfoxides [58, 59, 60, 61, 62, 63]. Around the same time Aggarwal first employed Hoppe lithiation of carbamates (X = OCb) or sterically hindered benzoates (X = OTIB) in the presence of Sparteine (**sp**) [64, 65, 66, 67, 68] or its surrogate [69]. Although the products of these lithiations can be inserted into boronic esters directly, it can be beneficial to isolate and store them as stannanes [69, 70, 71, 72, 73]. This is particularly useful as recrystallization can allow for increasing their enantiomeric purity to more than 99% ee. This helps to avoid the formation of diastereomers in iterative sequences. In situ reaction with butyl lithium reliberates the chiral carbenoid, which inserts cleanly into boronic esters (**17**→**20**).

**SCHEME 5:**
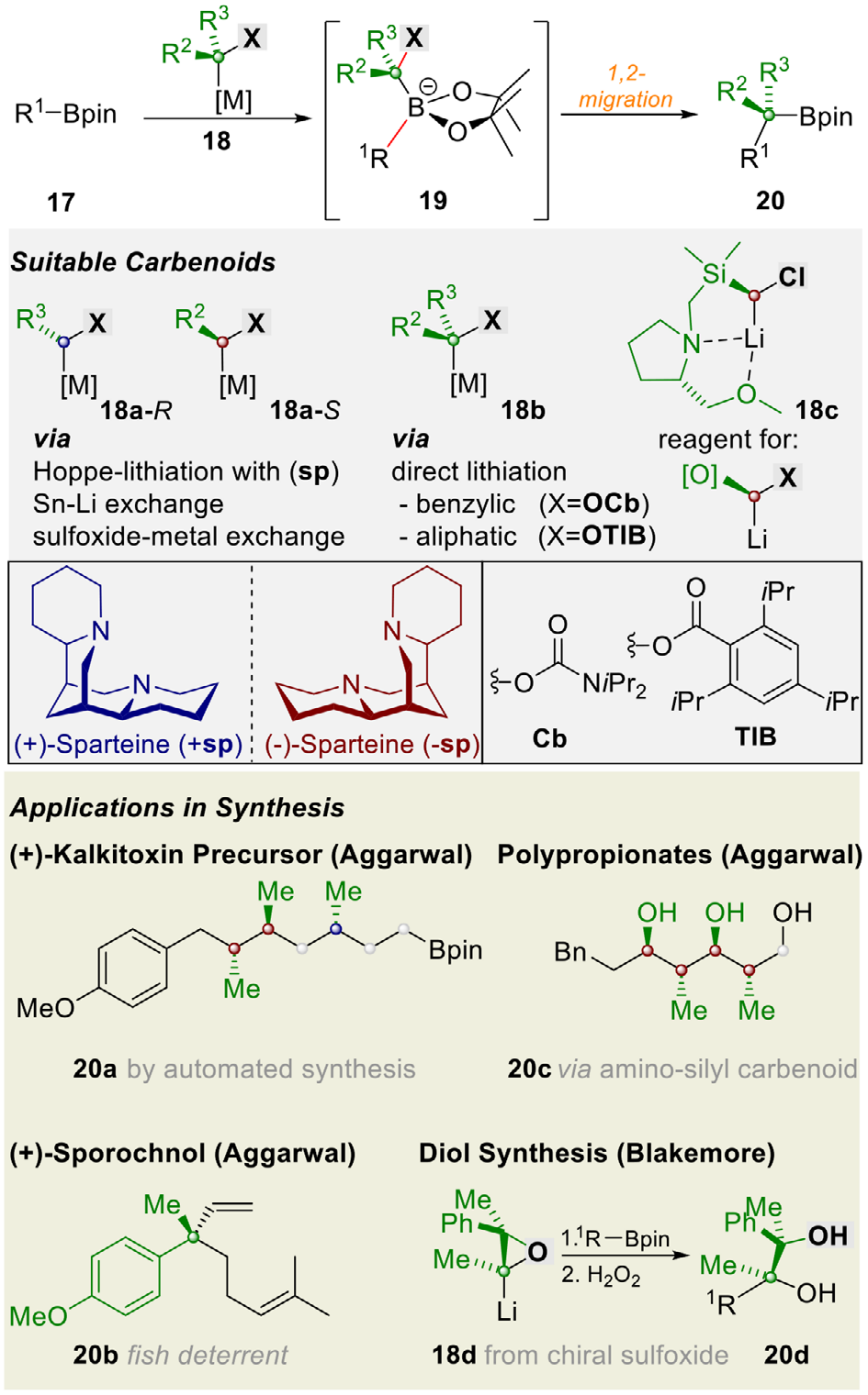
Stereospecific Reagent Controlled Homologation (StReCH) [59, 74, 75, 76].

This highly efficient reaction allows for conducting multiple homologations in the same pot. Due to this efficiency, Aggarwal coined the term “assembly‐line synthesis.” The (+)‐kalkitoxin precursor **20a** was synthesized by a fully automated robot, which conducted all six homologation reactions without intermittent workup [74]. While this impressive example relies on simple carbenoids of type **18a** (with R^1/2^ = H or CH_3_), more elaborate and even disubsitituted carbenoids can be inserted as well. Benzylic carbamates (OCb) or aliphatic benzoids (OTIB) derived from secondary alcohols can be lithiated to generate carbenoids of type **18b**. Insertion of such a carbenoid into a suitable boronic ester and subsequent Zweifel‐Olefination was used by Aggarwal to construct the quaternary stereocenter in the fish‐repellent Sporochnol (**20b**) [76]. Steric congestion of carbenoids of type **18b** can limit their stability/availability. This can be mitigated by coordinating groups (e.g. CH_2_Bn), which stabilize the carbenoid [14]. However, steric congestion also hampers formation and rearrangement of ate complexes of type **19**. This can be alleviated by converting the boronic ester of type **17** into a dimethylborane with a methyl Grignard reagent [71]. As the migration tendency of the CH_3_ group is comparably low, selective insertion into the bond between the boron atom and R^1^ can be achieved. For the introduction of masked heteroatom substituents Aggarwal developed the proline derived chiral silylcarbenoid **18c** [75]. The silane moiety in **18c** serves as a masked hydroxyl group, which Aggarwal exploited in the synthesis of polypropinate **20c**. In order to introduce hydroxyl groups on the first carbon atom (C1’) of the newly introduced substituent, lithiated epoxides can be employed [77, 78]. Direct lithiation of monosubstituted epoxides can be achieved with high diastereoselectivity. Higher substitution patterns, however, can lead to selectivity problems or thwart lithiation entirely [79]. Blakemore generated highly substituted lithiated epoxides such as **18d** through sulfoxide metal exchange. Insertion into homobenzyl pinacol boronic ester and subsequent oxidation to **20d** proceeded in 59% and 96% de [59].

### 1.3 Catalyst Controlled Stereoselective Boronate Homologations

Reagent controlled StReCH‐reactions require one equivalent of chiral material per homologation step. Although chiral diamines such as sparteine, can often be recovered after lithiation, catalytic homologation reactions have an obvious appeal.

In 1997 Jadhav and coworkers described the reaction of Cl_2_CHBpin with BuLi in the presence of Yb(III) and a chiral bisoxazolin ligand [80]. While several equivalents of the chiral ligand were necessary to achieve ee‐values >80%, this work formally remains the first example of a catalytic MH. Not until 2016, Fu and coworkers described a more widely applicable catalytic variant of this reaction (Scheme 6). A classic MH of a pinacol boronic ester (**17**) delivers racemic α‐chloro pinacol boronates of type **21**. Substitution with alkyl zinc reagents in the presence of a nickel catalyst and a chiral diamine ligand delivers substitution products enantioconvergently. In some cases, the results could be further improved by the addition of sodium iodide. The proposed mechanism is illustrated in Scheme 6 and starts by transmetallation to the R‐Ni^I^‐L* complex. Oxidation to R‐Ni^II^‐L results in cleavage of the C─Cl bond in **21** to an α‐boryl radical [81, 82]. Its reaction with the R‐Ni^II^‐L complex is likely to be the stereoselective step and completes the oxidative addition to **23**. Reductive elimination produces **22** and the Ni^I^‐catalyst, thus completing the catalytic cycle. The protocol allows for a broad range of alkyl zinc reagents to be coupled efficiently with α‐chloro boronic esters. The introduction of primary alkyl **22a** substituents, including ones carrying masked hydroxyl groups (**22c**) was successful. Secondary boronic esters are also tolerated, which allowed for iterative application (**22c**) of the process. Unfortunately, using secondary alkyl zinc reagents result in rather low yields and moderate *ee*s.

**SCHEME 6:**
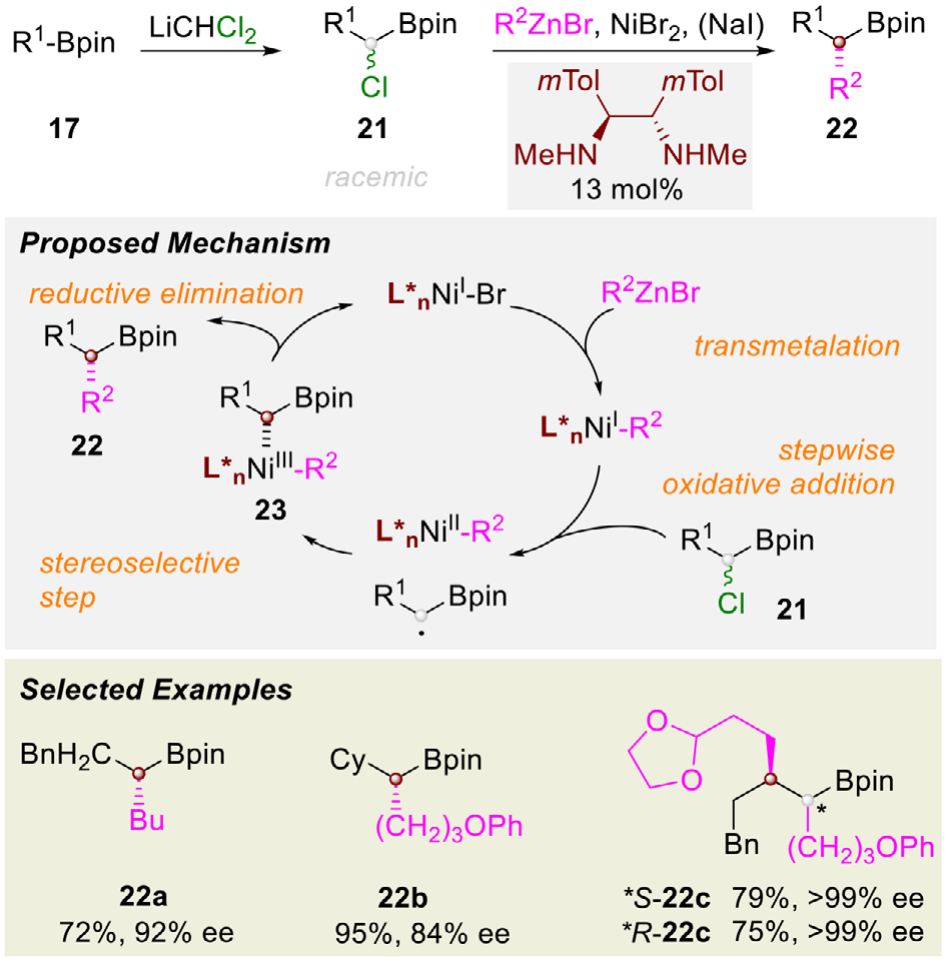
Fu´s enantioconvergent Ni‐catalytised Matteson‐substitution.

More recently the Jacobsen group presented a catalytic version of the Matteson homologation itself. Stereoselective homologation of pinacol boronates of type **17** with LiCHCl_2_ was achieved with two types of thiourea‐based chiral catalysts (Scheme 7) [83, 84, 85]. Pre‐catalysts of type **27a** perform well with smaller migrating groups, whereas pre‐catalysts of type **27b** are better suited for bulky migrating groups [84]. The pre‐catalysts are converted to their active forms, 27a* and 27b*, by treatment with bromochloromethyl pinacol boronic ester and *n‐*BuLi. These catalysts facilitate 1,2‐migration of R^1^ by lithium‐assisted abstraction of one of the two enantiotopic chloride‐atoms from **24**. In the favored transition state **25^‡^** the uninvolved chloride atom (highlighted in gold) is orientated away from the catalyst. If it would be orientated toward the catalyst the undesired enantiomer *ent*‐**25** would be formed. DFT‐calculations suggest that in the corresponding undesired transition state *epi*‐**25^‡^** orientation of said chloride atom (highlighted in gold), is not only sterically disfavored. It also competes for Li‐binding sites with the chloride atom, which is acting as a leaving group. All in all, **25^‡^** is favored over *epi*‐**25^‡^** by more than 5 kcal/mol (for R^1^ = *i*Pr), which allows for achieving impressive *ee*‐values (R^1^ = prim. 52%‐88% yield and ≥94% *ee*, R^1^ = sec. 63%‐74% yield and ≥89% *ee*). Analogous to α‐chloro boronates obtained by classic Matteson homologations (c.f. Scheme 2, **4→5**), **25** can undergo substitution with a variety of nucleophiles, including C‐, O‐, and N‐nucleophiles [83]. Unfortunately upon iterative application substrate control can become dominant over ligand control as apparent by comparison of examples **26a‐d**.

**SCHEME 7:**
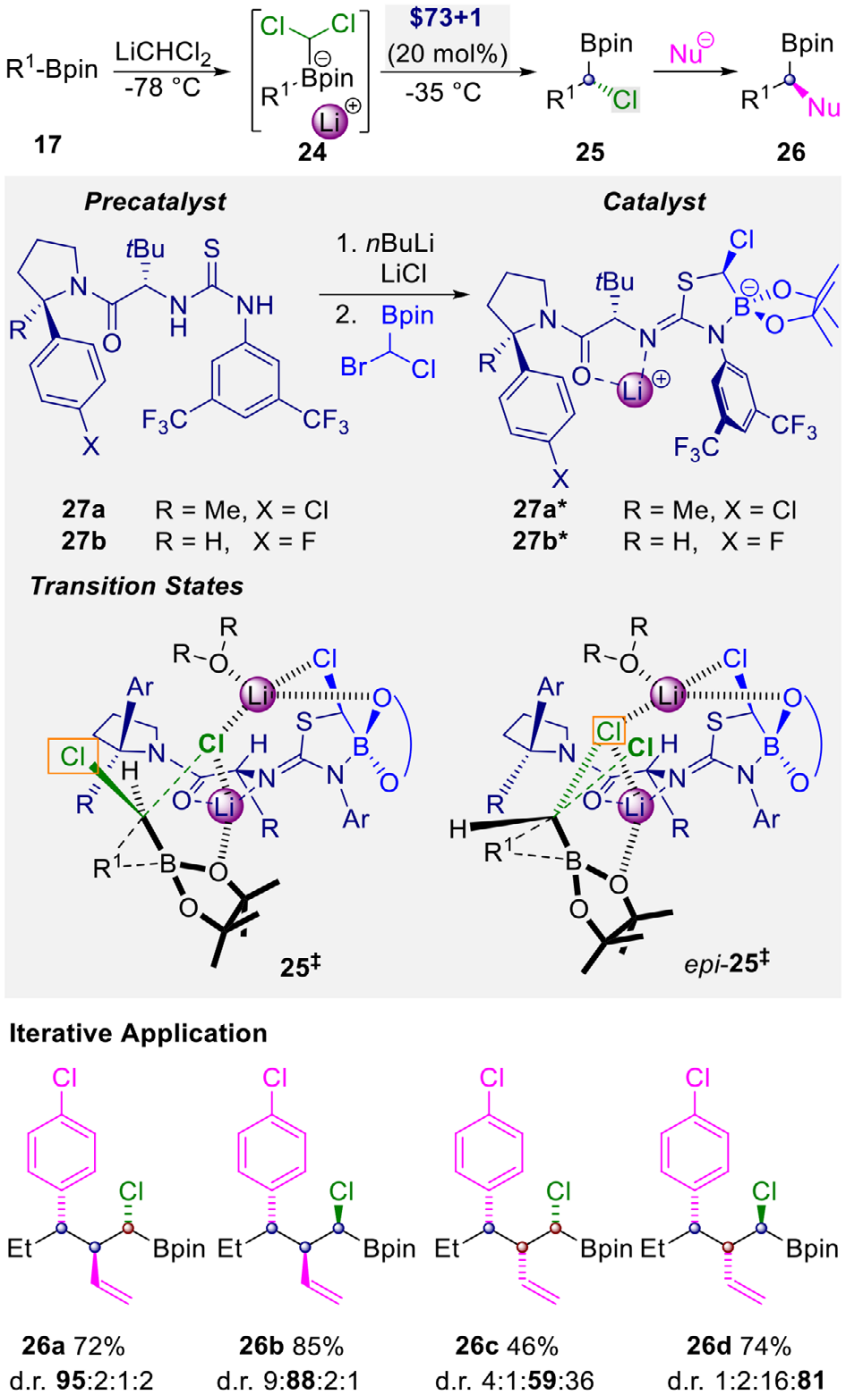
Jacobsen´s Li‐thiourea catalysed Matteson homologation.

### 1.4 Comparing the Different Approaches

To conclude the first part of this review all three approaches discussed are compared (Scheme 8). Substrate controlled boronate homologations, such as the classic Matteson‐Homologation (MH) and Dong´s new stereoconvergent homologation of chiral boroxazolidines rely on chiral auxiliaries (Section 1.1). In iterative syntheses, however, this is less of an issue then one might expect, as each new homologation benefits from the same chiral auxiliary. Both methods allow for the introduction of protected heteroatom substituents but are somewhat limited in terms of stereochemical variability by the chiral auxiliary. Furthermore, tertiary boronates used to be unavailable by classic MHs. Reagent controlled reactions, such as Aggarwal´s *assembly line synthesis*, offer a maximum of stereochemical diversity and can allow for the introduction of disubstituted carbenoids (Section 1.2). Due to the nature of chiral carbenoids, however, the introduction of masked heteroatoms is currently still limited to silanes, which can be oxidized to alcohols [75, 86, 87]. Furthermore, one equivalent of chiral carbenoid is necessary for each homologation. Fortunately, chiral diamines such as Sparteine can usually be recovered after lithiation.

**SCHEME 8:**
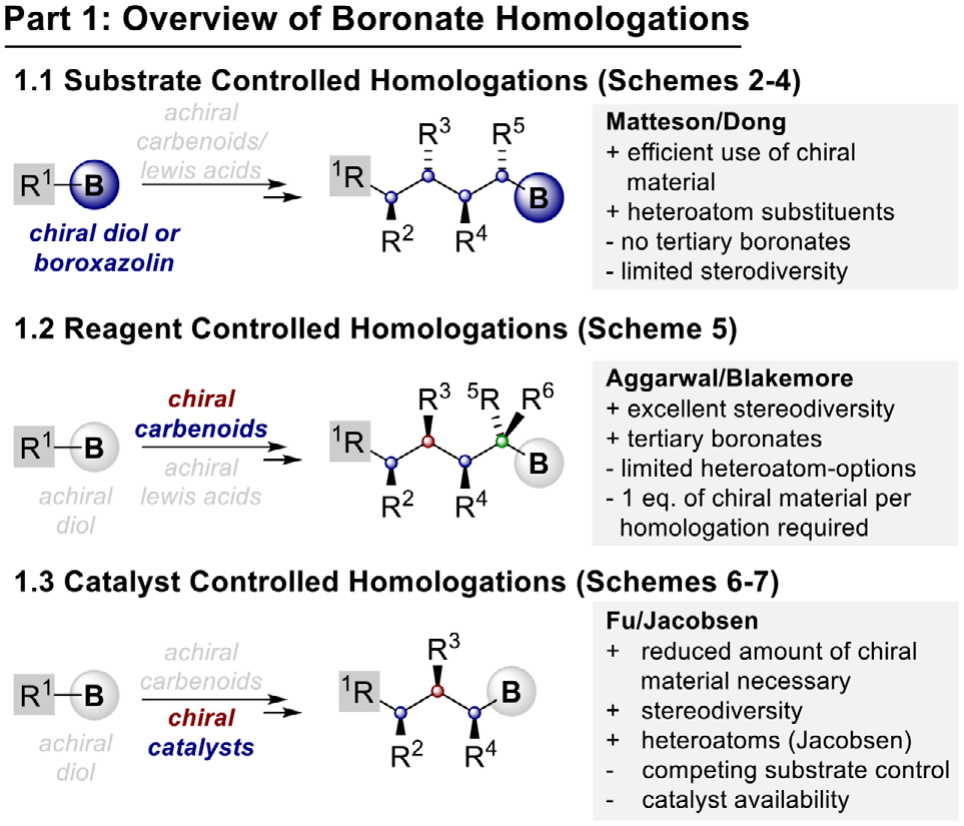
Synopsis of Part 1.

Finally, catalyst‐controlled reactions bear the promise of a more efficient use of chiral material and the last decade saw two impressive methods (Section 1.3). Fu established a diastereoconvergent substitution reaction, while Jacobsen achieved stereoselective insertion of LiCHCl_2_ into a pinacol boronic ester by catalysis. This allows for the introduction of numerous substituents as nucleophiles, similar to a Matteson‐reaction. Competing substrate control currently remains an issue.

Thus, while the pioneering work of Fu and Jacobsen is certainly impressive, substrate and reagent‐controlled methods still remain as the important work horses for state‐of‐the‐art boronate homologations. However, both of these approaches come with their own advantages and shortcomings. StReCH maximizes stereochemical flexibility. This, however, comes at a cost, as each iteration requires the use of one equivalent of chiral material. In iterative Matteson‐sequences on the other hand, the cheap chiral auxiliary can perform its function over and over again. Therefore, methods and strategies that allow for diverging from the configuration dictated by the chiral auxiliary are of high synthetic value. Recent years have seen some interesting developments in this field. These developments as well as some classical solutions for this problem are discussed in the second part of this review.

## 2 Stereodivergence in Substrate Controlled Homologations

Until the arrival of Dong´s enantioconvergent homologation (Scheme 4), substrate controlled boronate homologations were basically synonymous with Matteson homologations (Scheme 2). For this reaction two chiral auxiliaries have emerged, which usually overwrite the influence of all other stereogenic centers. Thus, these auxiliaries are often also referred to as chiral directors. Pinanediol was first introduced and can be synthesized by Upjohn bishydroxylation of pinene [39], both enantiomers of which are cheaply available. Pinanediol boronates are highly stable, which makes them easy to handle [18, 88]. Both enantiomers of DICHED (**DiC**yclo**H**exyl**E**thane**D**iol) can be prepared by Sharpless bishydroxylation of Stilbene and subsequent hydrogenation [89, 90]. DICHED‐boronic esters are also conveniently stable. Furthermore the director provides additional stereodifferentiation and thus often improved diastereoselectivity [40]. Compared to the boronic esters used by Matteson, Dong´s boroxazolidines are of limited stability [56]. While this might seem less than ideal for longer sequences, it also opens up convenient pathways toward stereodivergent outcomes. In this section we discuss different ways to increase the stereochemical variability in iterative homologation sequences, starting with a simple exchange of the chiral auxiliary.

### 2.1 Change of Chiral Auxilliary

When divergence from the configuration dictated by an auxiliary is desired in an iterative sequence, its replacement is the most straight forward solution.

However, hydrolysis of cyclic boronates is entropically disfavored. Therefore, some means of shifting the equilibrium in favor of the boronic acid is required. As shown in Scheme 9A, this can be achieved by destruction of the diol with NaIO_4_ [91, 92] (**28**→**29**). Due to the harsh nature of the oxidant, this method is not broadly practicable. If the boronic acid is sufficiently water soluble, transesterification with phenylboronic acid in a biphasic system, can be employed (Scheme 9B) [91, 92]. Pinanediol boronic esters can also be cleaved by refluxing with LiAlH_4_. This leads to a pinanediol‐alanate complex that can be precipitated, as well as the corresponding borohydride, which can later be hydrolized [93, 94]. Another way of shifting the equilibrium by crystallization is shown in Scheme 9C. It relies on conversion of the boronic ester into the corresponding trifluoroborate (c.f. **31**). If this allows for crystallization, good yields can be achieved [95]. For larger substrates, not prone to crystallization, a more recent approach reported by Klein and coworkers might be more suitable (Scheme 9D). It exploits the volatility of methylboronic esters such as **32a**, which are formed by transesterification after addition of methylboronic acid. The volatile methyl boronic ester **32a** can be removed from the equilibrium by heating under reduced pressure [96]. The method tolerates a wide range of functional groups, including heterocyclic and vinylic compounds, as well as both base‐labile (Fmoc) and acid‐labile (Boc) protecting groups.

**SCHEME 9:**
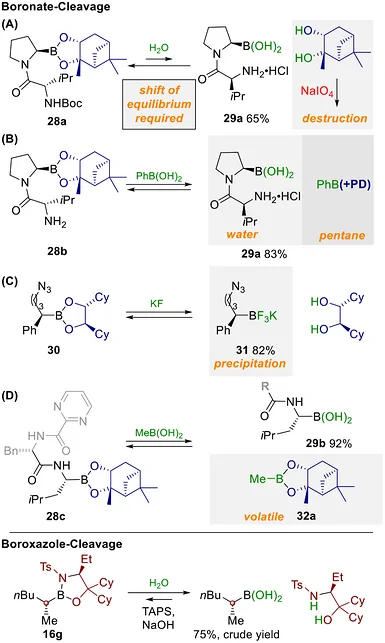
Hydrolysis of boronic esters requires additional means for shifting the equilibrium, while Boroxazoles hydrolyze readily.

While hydrolysis of DICHED‐ and pinanediol‐boronic esters requires some effort, boroxazolidines such as **16** **g** hydrolyze swiftly in biphasic systems. This lower thermodynamic stability allows for a simple change of chiral director, but also makes workup and purification more difficult [97].

However, all changes of chiral directors that rely on cleavage and reattachment prolong synthetic sequence by two steps. Fortunately, differences in thermodynamic stability allow for direct transesterifications as well. As depicted in Scheme 10, transesterification of unstable boroxazolidines such as **14**‐*R* to pincacol boronic esters **17** proceeds readily. Such tranesterifications were conducted by Dong and coworkers directly before the work‐up of a homologation reaction. Pinacol boronic esters (**17**) on the other hand can be transesterified to DICHED boronic esters (**33**) in Et_2_O [98]. Thus, there is no reason to suspect that a direct change from a boroxazolidine of type **14** to corresponding DICHED boronic esters (**33**) should not be possible. Since Dong´s method has been published less than a year before the writing of this review, no such direct transesterification has been reported. However, such a reaction could serve to combine Dong´s and Matteson´s methods in an iterative homologation sequence. This would allow for a change of chiral director without an additional purification step.

**SCHEME 10:**
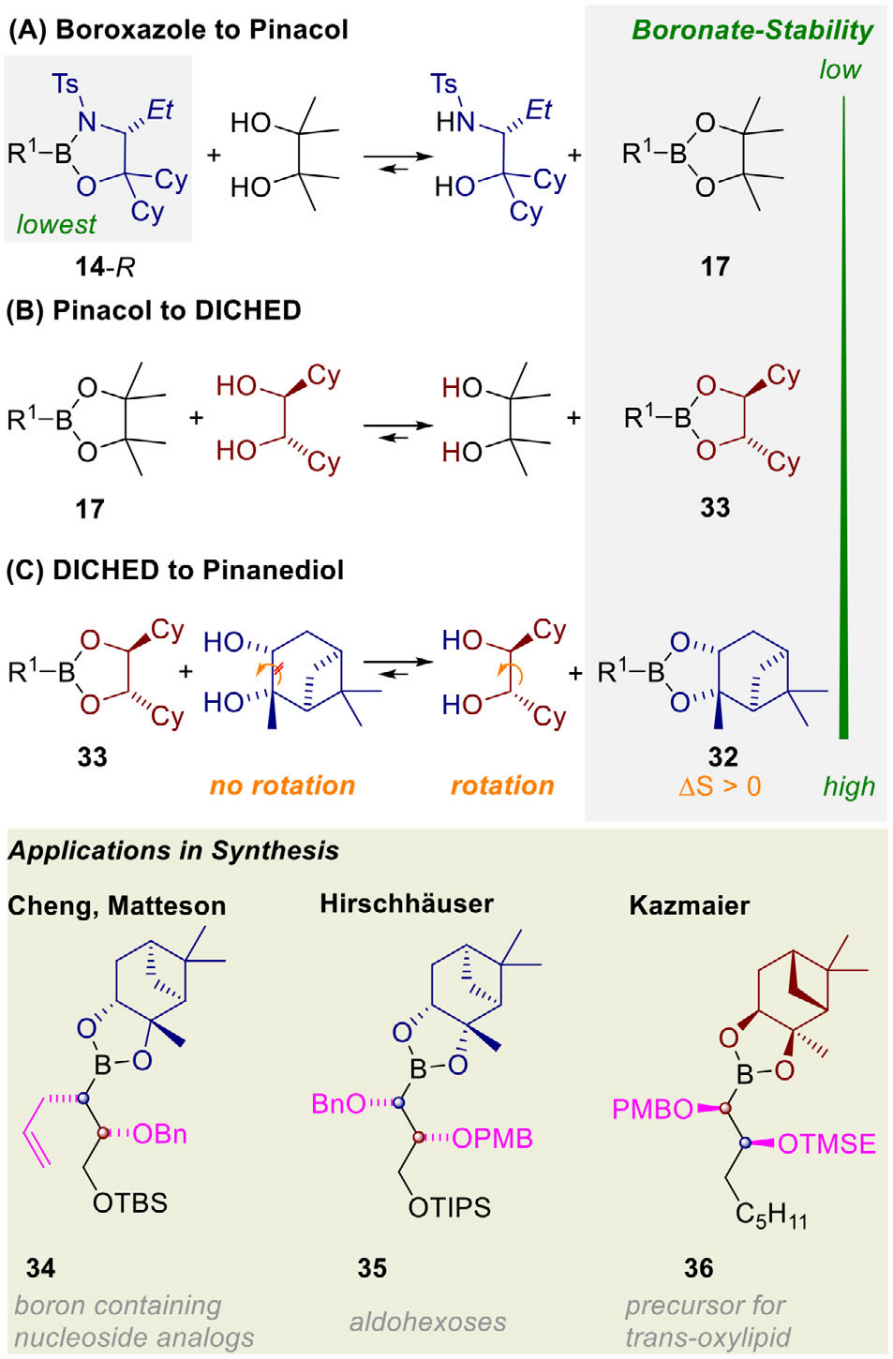
Transesterifications of chiral auxiliaries and their application in synthesis.

A change of DICHED to pinanediol on the other hand has been employed by Cheng and Matteson to prepare intermediate **34**, for their synthesis of boron containing nucleoside analogs [88]. It is particularly useful for the generation of *threo*‐glycols such as **35**, which we required for our synthesis of aldohexoses [43]. A key driving force of this facile reaction is the increase in enthropy. It can be attributed to additional rotational freedom around the glycol C─C‐bond in DICHED, which is gained after its liberation from the boronic ester. In return no enthropy is lost upon esterification of pinanediol, due to its rigid structure (c.f. Scheme 10C). When the reaction is conducted in Et_2_O, DICHED often precipitates, which helps to drive the reaction to completion. This, however, can take several hours and can be hampered by impurities. By conducting a similar transesterification in pentane, Kazmaier and coworkers were able to complete the transesterification in 2 h at room temperature and reliably reclaim DICHED by precipitation. In their synthesis of *trans*‐oxylipids, the group employed this strategy for the synthesis of *threo*‐glycol **36** [99].

These reports show that at least one exchange of a chiral director within a synthesis based on iterative Matteson‐reactions can be achieved quite easily by transesterification of DICHED to pinanediol. If transesterification of boroxazolidines to DICHED proceeds as easily as to pinacol, a second change of auxiliary should also be straight forward.

### 2.2 Strategic Solutions

In addition to changing the chiral auxiliary, there are several strategies for mitigating the stereochemical limitations of MHs by synthetic planning. For carbon nucleophiles, divergent relative configurations can be obtained by swapping the role of the nucleophile and the boronic ester. Matteson showed this using the simple model shown in Scheme 11A. It showcases a homologation in which the introduction of a methyl‐substituent is desired (**37** to **38**) [40].

**SCHEME 11:**
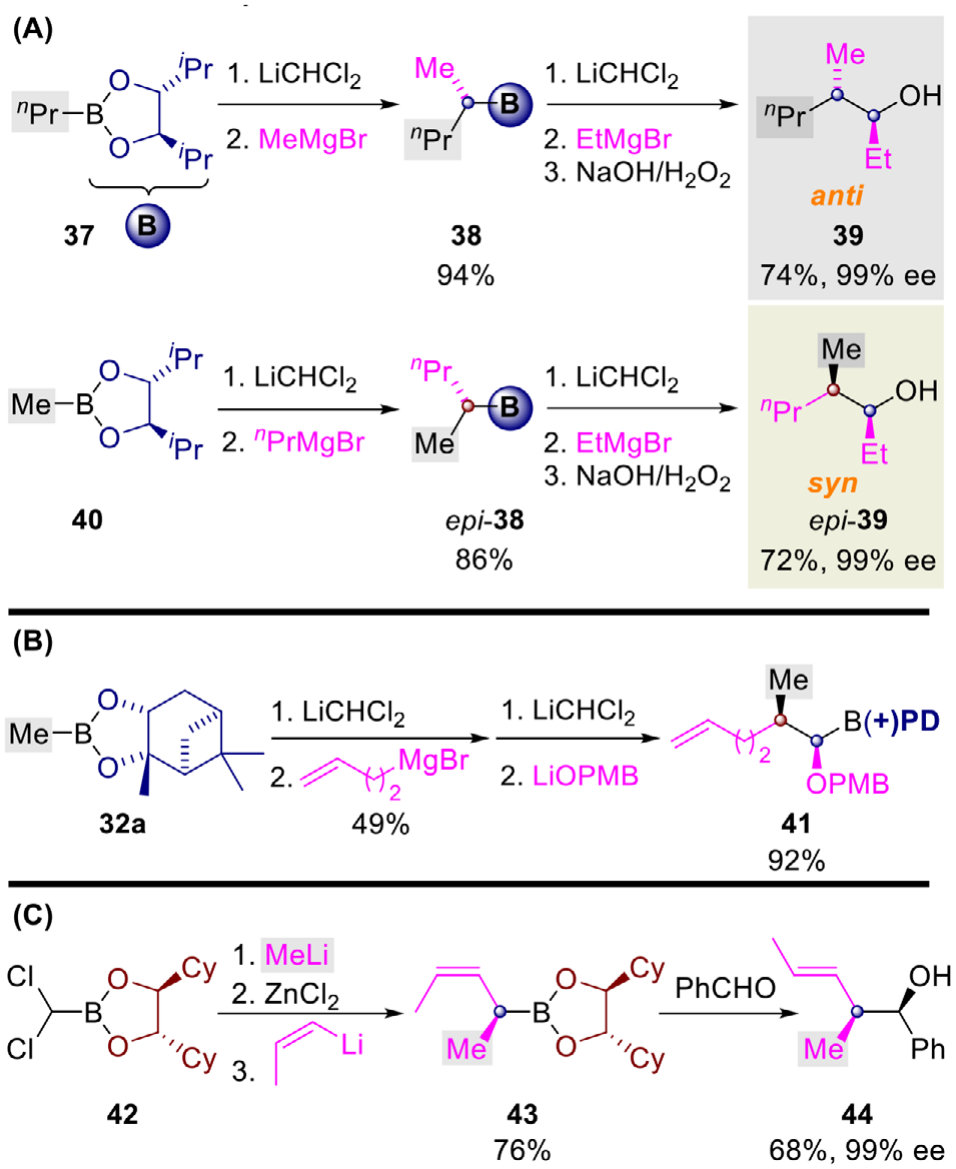
Strategic choice of starting materials.

DiIsoPropylEthaneDiol (*R,R)*‐DIPED is an older variant (*R*,*R*)‐DICHED and can be employed in a similar manner. Propylboronic ester **37** undergoes two standard Matteson reactions, introducing methyl (**38**) and ethyl substituents. Subsequent oxidation furnishes alcohol **39**, in which the methyl and ethyl substituent are in an *anti*‐relationship. To access the corresponding *syn*‐diastereomer *epi*‐**39**, the sequence is initiated with the methylboronic ester **40**. The isopropyl group is installed as the nucleophile during the first homologation to obtain the boronate *epi*‐**38**. Subsequent homologation, introduction of the ethyl group and oxidation furnishes the *syn*‐isomer *epi*‐**39**. Both diastereomers were obtained with high yields (74% and 72%) and *ee*s (99%). The Armstrong group showed that methyl pinandiol boronic esters such as **32a**, can be used in a similar way (Scheme 11B) [100]. An even more versatile reagent is dichloromethyl boronic acid DICHED ester **42** (Scheme 11C). Due to its C_2_‐symmetry its reaction with a Grignard reagent of type R^1^MgX leads to the same ate complex as the reaction of a corresponding boronic ester (i.e., R^1^‐B(DICHED)) with LiCHCl_2_. Addition of propenyl lithium leads to the homologation/ substitution product **43**, which itself is a useful reagent for the allylation of aldehydes (**44**). Thus, the configuration of the first stereocenter in an iterative Matteson synthesis can be chosen independently of the chiral director. This can be achieved either by varying the order in which organometal reagents are reacted with **42**, or by selecting an appropriate boronic ester as starting material. The limitation of this approaches become apparent when compared to the transesterification method. Only the first stereocenter can be varied this way, whereas subsequent iterations remain controlled by the chiral director. Furthermore, the scope of the approach is limited to systems in which both substituents are carbon‐based. The extension of this strategy to heteroatom‐containing substituents remains underdeveloped, as this would involve homologation of borates, of which only few examples have been reported [10, 11].

A second chance to choose a configuration independently of the chiral director is available at the end of an iterative synthesis based on MHs. The strategy relies on different conversions of the C─B bond. We applied this in our aldohexose synthesis (Scheme 12A). Following homologation and substitution of boronate **45** with Lithiumbenzoxide, Zweifel olefination of the boronic ester **46** furnished the masked *erythro*‐glycol *erythro*‐**47** [43]. Alternatively, **45** can be homologated and reacted with vinyl Grignard. Oxidation and O‐benzylation yields the corresponding *threo*‐glycol *threo*‐**47**. In the first case, the alkoxy group was introduced as a nucleophile and the boronate was converted into a vinyl group. In the second case the vinyl group was introduced as a nucleophile and the boronate was converted into the alkoxide. Thus, by varying the final conversion of the boronic ester, the configuration of the last stereocenter in an iterative Matteson synthesis can also be selected independently of the chiral director. Key to this strategy is the ability to convert C─B into C─C‐bonds.

**SCHEME 12:**
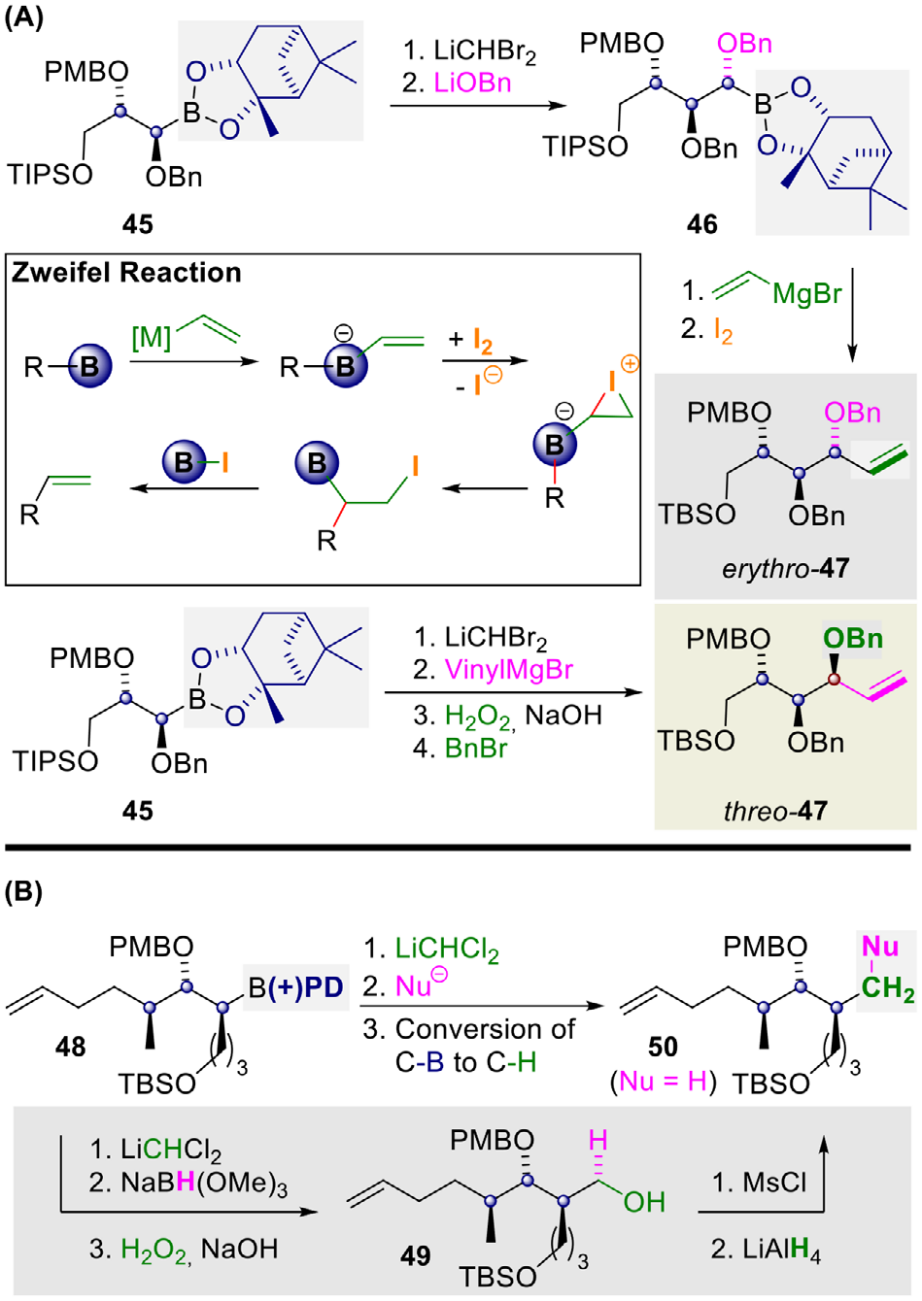
Strategic choice of sequence termination.

If an alkyl‐ instead of a vinyl substituent is required, a Matteson‐sequence can be used for this (Scheme 12B). This was shown in Armstrong's synthesis of a tautomycin fragment, in which boronate **48** was homologated using LiCHCl_2_, substituted with NaHB(OMe)_3_ and oxidized to **49**. To remove the remaining alcohol moiety, **49** was first mesylated and then reacted with LiAlH_4_ to **50** [100]. Since secondary alcohols can be removed in a similar manner, Grignard reagents could be used instead of NaHB(OMe)_3_. This would allow for the introduction more diverse alkyl‐chains.

Due to the multitude of functional group interconversions available for boronic esters [101], this approach could be quite versatile. One of the most well‐known methods in organic synthesis for transforming C─B into C─C bonds is the Suzuki–Miyaura cross‐coupling (SMC). It requires activation of the boronic ester with a base. The resulting ate complex can then undergoe transmetallation. SMC of α‐chiral pinacol boronic esters with aryliodides has been reported to proceed under retention [102, 103, 104]. While there are no examples of α‐chiral DICHED or pinanediol boronic esters, SMC of vinyl pinanediol boronic esters has been reported [105, 106]. Thus it is not unfeasible to suspect that a sequence of MHs could be concluded with a SMC that proceeds under retention of the configuration. However, not every reaction that relies on turning a boronic ester into a nucleophile proceeds under retention. As shown in Scheme 13, converting a sp^3^‐hybridized pinacol boronic ester into a carbon nucleophile can be employed for a variety of transformations. As in a SMC the boronic ester must first be converted into an ate complex of type **51** by reaction with a nucleophile. Depending on the conditions subsequent reaction with an electrophile can proceed either under inversion or retention.

**SCHEME 13:**
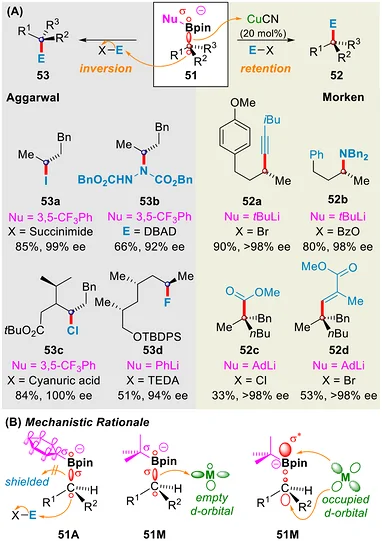
Transformation of activated pinacol boronic esters.

Morken's group treated secondary pinacol boronic esters with *tert*‐butyllithium to generate activated ate complexes of type **51**. In the presence of a catalytic amount of CuCN, these ate complexes underwent stereospecific transmetalation under retention. The resulting cuprate was then reacted with a variety of different electrophiles to furnish products of type **52** under retention. The reaction exhibits a broad substrate scope, accommodating unsaturated carbon electrophiles, acyl chlorides, β‐haloenones and hydroxylamines. For example, alkynyl bromides were reacted to **52a** in very high yield and >98% ee. Transfomation into debenzylated amine **52b** was achieved with hydroxylamine in the presence of CsF in 80% yield and 98% ee. In general, the reaction proceeds in high yields and with good stereochemical fidelity [107]. In later work, Morken extended this transformation to tertiary boronic esters. Akin to the previous method, the boronic ester is activated via formation of ate complex **51** albeit this time with adamantlylithium. Transmetallation to copper and subsequent reaction with electrophiles furnishes coupling products of type **52**. Methyl ester **52c** was formed by the reaction with methyl chloroformate with a rather low yield. Treatment with β‐haloenones and hydroxylamine gave the synthetically valuable α,β‐unsaturated ester **52d**. Compared to the secondary boronic esters, the yields are generally lower [108]. Aggarwal also employed nucleophilic ate complexes of type **51** generated by adding aryllithiums to secondary pinacol boronic esters. However, direct reaction with a variety of electrophiles furnished substitution products of type **53** under stereochemical inversion! This requires the electrophile to attack the small lobe of the C‐B‐σ‐orbital shown in red. This S_E_2*inv* mechanism leads to inversion. *N*‐Iodosuccinimide (NIS) can be used to generate iodides such as **53a**. Reaction with di‐*tert*‐butyl azodicarboxylate furnished hydrazine **53b**. Trichlorcyanuric acid allows for the introduction of chloride, even in the presence of a *t*‐Bu‐ester (**53c**) [109]. Activation of secondary boronic esters with phenyllithium followed by treatment with Selectfluor II resulted in **53d**. Carbon electrophiles reported are limited to the cycloheptatrienylkation (not shown) and the reaction does not tolerate tertiary boronic esters [110].

So, the question arises, why ate complexes of type **51** react under inversion in one case and under retention in the other? As shown in Scheme 13B Aggarwal generated aryllithium derived ate complexes of type **51A** and reacted different electrophiles with them directly. Here the reaction with electrophiles takes place at the small lobe of the red C─B σ‐bond (i.e., the HOMO). Attack of an electrophile at the big lobe of the σ‐orbital is disfavored, due to shielding by the congested borate. The same is true for the pink C‐B‐bond to the aryl‐group, which is activated as well. However, attack on its small lobe is prohibited by the aromatic system, which leaves the depicted reaction as the only viable option. Morken´s reaction, relies on transmetallation of an ate complexes of type **51** **M**, in which *t*BuLi was used for activation. In this case the metal has to approach the large σ‐lobe of the red C─B bond from the side. This way π‐back donation from an occupied d‐orbital into the σ*‐orbital can assist cleavage of the C─B bond. Again, this reaction competes with the equivalent interaction with the *t*Bu‐B bond (pink). This explains why only secondary boronic esters could be reacted using *t*BuLi for **Nu** (**52a/d**). In tertiary boronic esters insertion into the desired σ‐bond would be less favorable than into the *t*Bu‐B bond. In such cases the sterically even more demanding adamantly‐Li had to be used for activation. A similar rationale can be applied to SMCs, which also proceed under retention.

These mechanistic considerations aside, we can conclude that there are many ways to vary the configuration of both the first and the last stereocenter introduced in an iterative MH‐based synthesis without any need for changing the chiral director.

### 2.3 Controlling Divergent Stereocenters in the Middle of an Iterative Sequence

Installing a stereocenter with a divergent configuration in the middle of an alkyl chain constructed by iterative MHs is somewhat more challenging. In recent years several new methods for this specific task have been developed. We will refer to these methods as *epi*‐Matteson reactions. They greatly expand the versatility of the MH and allow chemists to tailor stereochemical outcomes flexibly across complex, multifunctional targets.

Kazmaier and coworkers realized such an *epi*‐Matteson reaction by utilizing lithiated 1,1‐dihaloalkanes (X_2_CHR^2^) as carbenoid precursors. The resulting carbenoids were reacted with (*S,S*)‐DICHED boronic esters. The resulting α‐alkyl‐halo boronic ester was treated with a nucleophilic hydride source (LiBHEt_3_), yielding the epi‐Matteson product (Scheme 14A). To assess the substrate scope the boronates were oxidized with hydrogen peroxide under basic conditions (**55**) [111]. Primary alkyl boronates were converted into products such as **55a** with high yields and good *ee*s. A phenyl boronic ester, could also be converted with good diastereoselectivity (**55b**). Classic MH products of aryl boronic esters are notoriously prone to isomerization [73, 112, 113], which makes **55b** particularly valuable. Matteson products themselves are suitable substrates for this type of reaction, which was demonstrated by the synthesis of stereodivergent products such as **55c**. The nature of R^2^ on the dichloroalkane was varied as well so that an ethyl‐ (not shown) or *Z*‐but‐2‐enyl‐substituent (**55d**) could be introduced successfully. Insertion of lithiated Dichlor‐phenylmethane into homobenzyl‐pinacol‐boronic ester yielded **55e** after oxidation. Surprisingly this product was primarily formed in the normal Matteson‐configuration and thus deviates from the previously observed diastereoselectivity.

**SCHEME 14:**
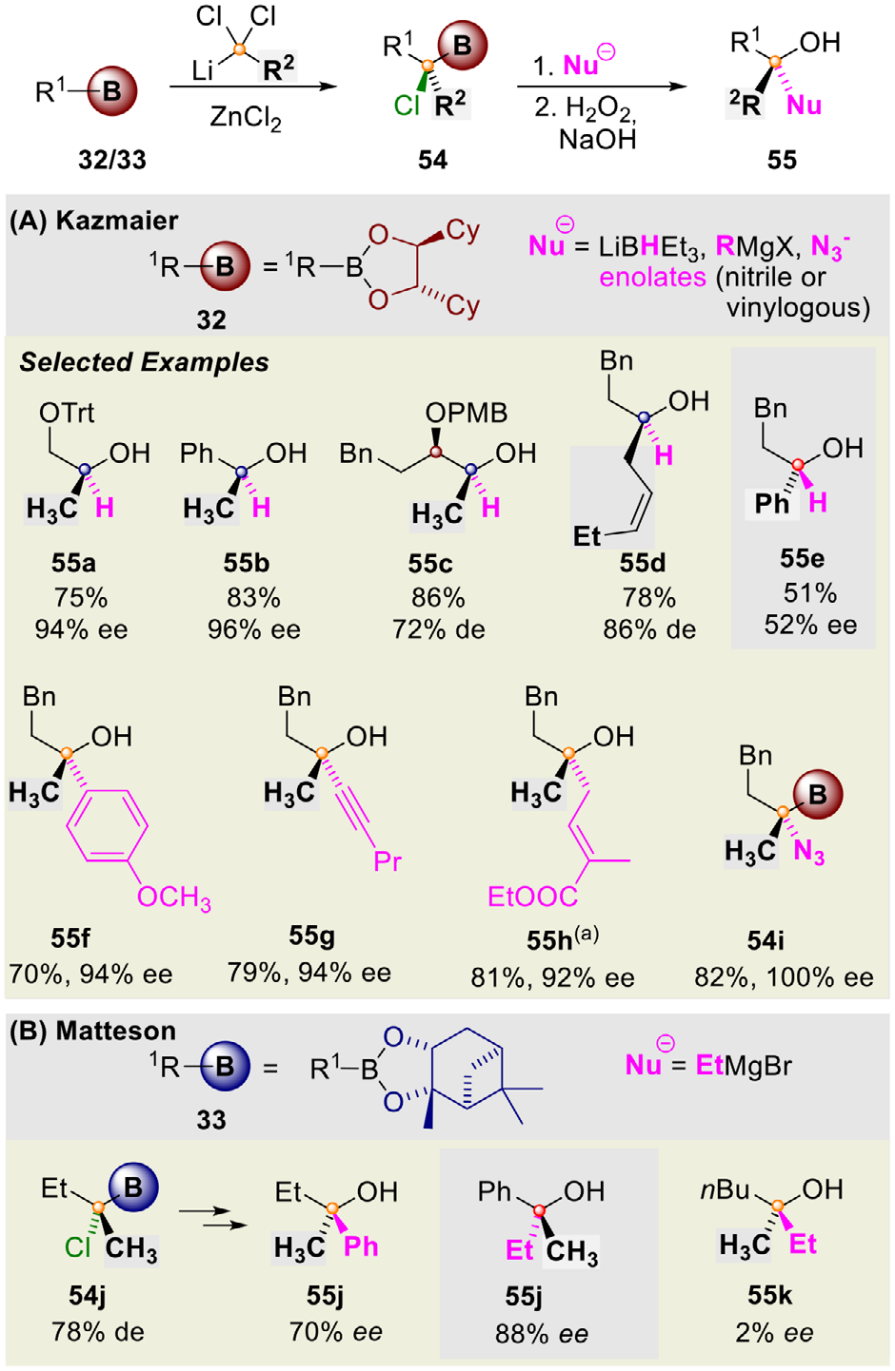
Homologation of boronic esters with lithiated 1,1‐dichloroalkanes. (a) Homologation conducted with LiBr_2_CCH_3_.

By reacting α‐chloro boronic esters of type **54** with other nucleophiles, such as Grignards, enolates or azide, other interesting boronic esters (or their oxidation products) can be obtained. Reaction with aryl and alkyne nucleophiles resulted in **55f** and **55** **g**. Various enolates were employed as well, including nitrile and vinylogous enolates (**55** **h**). Notably the use of azide allowed for the introduction of a masked amine yielding **54i**, which is an interesting precursor for chiral tertiary amines. Unfortunately, disubstituted α‐halo boronic esters of type **54** did not react with oxygen nucleophiles. Furthermore reacting **54** with *i*‐PrMgCl preferably led to O‐migration of the DICHED auxiliary (c.f. Scheme 2, *epi*‐**4**→**8a**).

To avoid this, one might be tempted to employ the corresponding pinanediol boronic esters (Scheme 14B). This had already been briefly investigated by Matteson and a coworker in 1990, in an attempt to establish a reliable pathway toward tertiary boronic esters [114]. (+)‐Pinanediolboronates of type **33** were reacted with lithiated 1,1‐dichloroethane to furnish α‐haloboronic esters such as **54j**. Substitution of **54j** with ethyl Grignard, followed by oxidation of the boronic ester, afforded the expected tertiary alcohol **55j** in 70% ee. By exchanging the role of R^1^ and Nu one would expect formation of the enantiomer (*ent*‐**55j**). However, starting the reaction with the phenylboronic ester and using ethyl Grignard for the substitution led to the same enantiomer of alcohol **55j** after oxidation. Moreover, upon switching to a butyl boronic ester almost no selectivity was observed at all (**55k**). Secondary boronic esters (*i*Pr and CyHex) were tested as well, apparently leading to α‐halo boronic esters with decent diastereoselectivity. However, their detailed assignment was limited by the analytical methods of the day. Thus, Matteson concluded *“that the diastereoselection in the migration of normal alkyl substituents longer than ethyl turns out to be uselessly poor as well as unpredictable in direction.”*

Given the encouraging results of Kazmaier, pinanediol boronic esters might deserve another look. Especially as similar stereodivergent outcomes upon substitution of benzylic α‐halo boronic esters were observed for both Kazmaiers DICHED (yielding **55e**), as well as Mattesons pinendiol boronates (yielding **55j**). While no mechanistic reason for this divergent stereoselectivity was reported, diastereoconvergent resolution of configurationally unstable α‐halo boronic esters is at least known to be possible. Matteson used the swift isomerization of racemic α‐iodo pinacol esters for the diastereoselective alkylation of Evans‐enolates [115]. More recently we applied the concept to α‐iodo pinanediol boronates to generate *epi*‐Matteson products as depicted in Scheme 15 [116].

**SCHEME 15:**
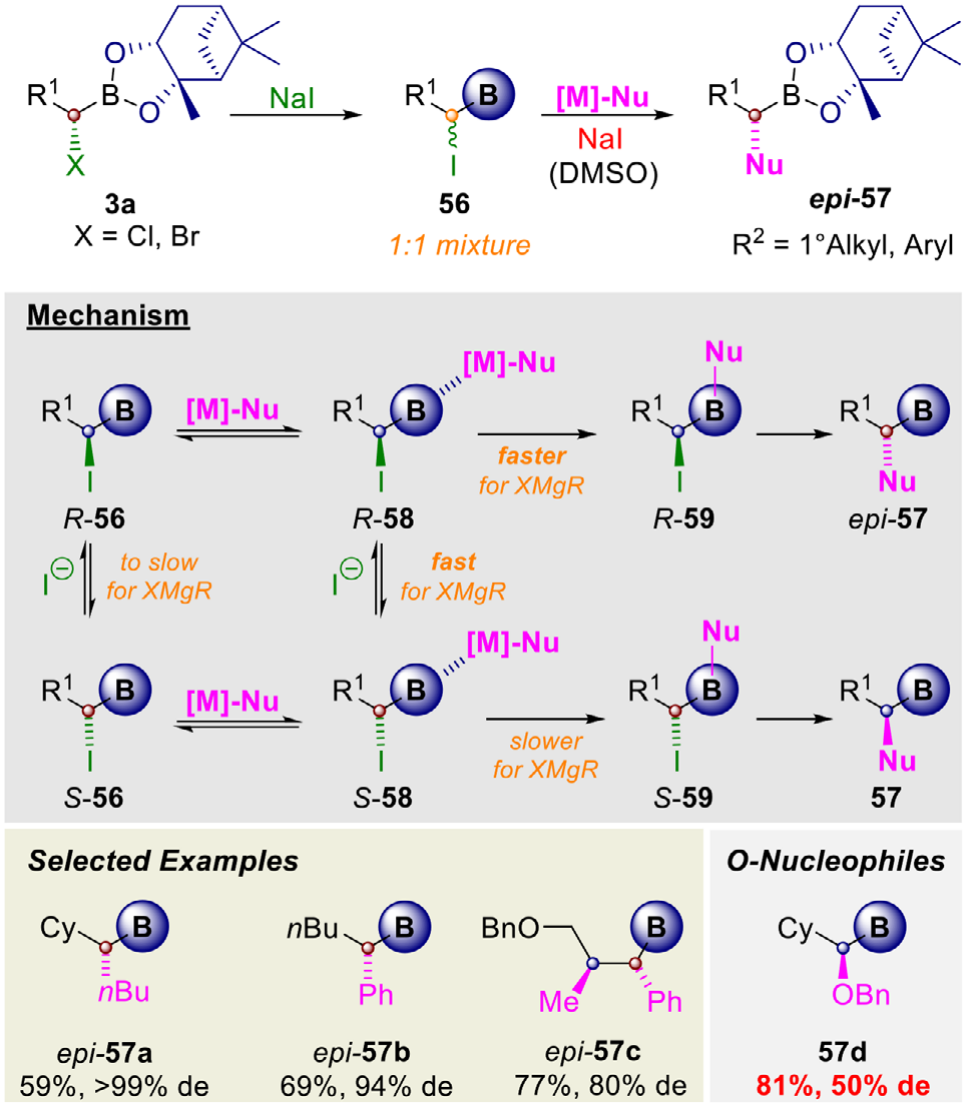
Diastereoconvergent *epi*‐Matteson Homologation with α‐iodo boronic esters.

Therefore α‐haloboronic esters of type **3a** were treated with sodium iodide to generate a 1:1 mixture of diastereomeric α‐iodoboronic esters **56**. Both diastereomers reacted to *epi*‐Matteson products of type *epi*‐**57** with primary alkyl Grignard‐ (*epi*‐**57a**) and aryl Grignard reagents (*epi*‐**57b** and *epi*‐**57c**). DFT calculations indicate that isomerization of **56** is accelerated by O‐Mg coordination to the Grignard reagent (**58**). This sets up a Curtin‐Hammett equilibrium from which the ate complex *R*‐**58** is preferentially formed. It then swiftly undergoes 1,2‐rearrangement to the *epi*‐Matteson product (*epi*‐**57**). Unfortunately, the protocol remains limited to phenyl and primary alkyl Grignard reagents. A disappointing but mechanistically interesting observation was made on the attempt to introduce alkoxides. This preferably delivered the classic Matteson products, albeit with a decreased diastereoselectivity (**57d**). Although this mechanistic oddity is still under investigation, it appears that ate complexes of type **59** are formed reversibly with alkoxides under these conditions. This makes the 1,2‐rearrangement from *S*‐**59** to **57** the rate‐determining step, which apparently favors the “classic” diastereomer **57**. Another disappointing observation was made when the reaction was attempted with *i*‐Pr‐Grignard, which did not deliver the desired substitution product. The most likely explanation for this failure lies in a competing iodine‐metal exchange reaction. As shown in Scheme 16, this process was exploited by Studer and coworkers to activate comparable α‐iodo DICHED boronic esters [117].

**SCHEME 16:**
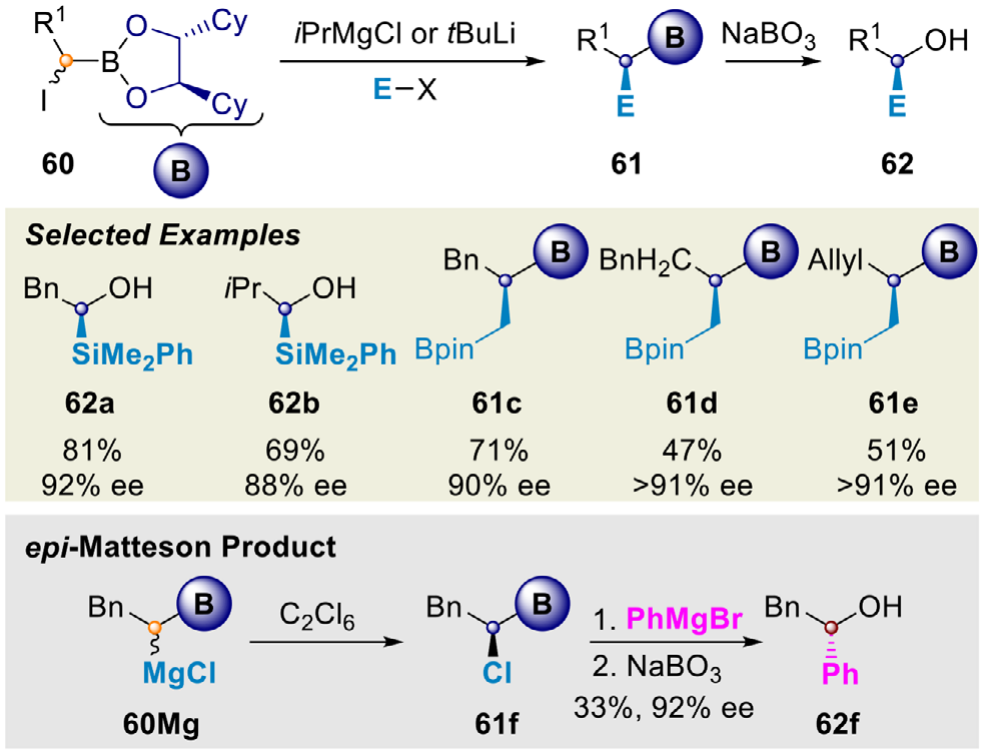
Electrophilic stereodivergent coupling by transmetallation of α‐iodoboronic ester.

Treating a diastereomeric mixture of α‐iodo DICHED boronates (**60**) with either isopropylmagnesium chloride or *tert*‐butyllithium led to iodine‐metal exchange. The resulting α‐metal boronic esters reacted diastereoselectively with hard electrophiles to standard Matteson products of type **61**. Silylation reactions were followed immediately by oxidation with NaBO_3_ yielding **62a** and **62b** with high 92% and 88% *ee*, respectively. These products are hard to obtain by classic MH, as nucleophilic silicon reagents are difficult to handle [118, 119, 120] and can require the use of lithium metal [121, 122]. By quenching with BrCH_2_Bpin alkylation was realized (**61c‐e**). Unfortunately, boronic esters containing simple, primary alkyl substituents delivered less satisfactory diastereoselectivities. With regard to *epi*‐Matteson reactions one particularly intriguing experiment was described. After iodine‐magnesium exchange to metalate **60Mg** hexachloroethane was added. This generated the α‐chloroboronic ester **60f** exhibiting the opposite configuration to that of the standard MH product. Subsequent treatment with phenyl Grignard delivered *epi*‐Matteson product **62c** with 33% yield and 92% *ee*. The low yield in this case is in line with Matteson´s observations that chlorides such as **60f** preferably undergo O‐Migration, when reacted with Grignard reagents (c.f. Scheme 2). As the more rigid pinanediol scaffold is less prone to this type of ring expansion. Thus the question arises whether pinanediol esters of type **56** might lead to better results here?

A currently more reliable pathway to *epi*‐Matteson products as well as tertiary boronic esters is provided by StReCH reaction with appropriate boronic esters (Scheme 17A).

**SCHEME 17:**
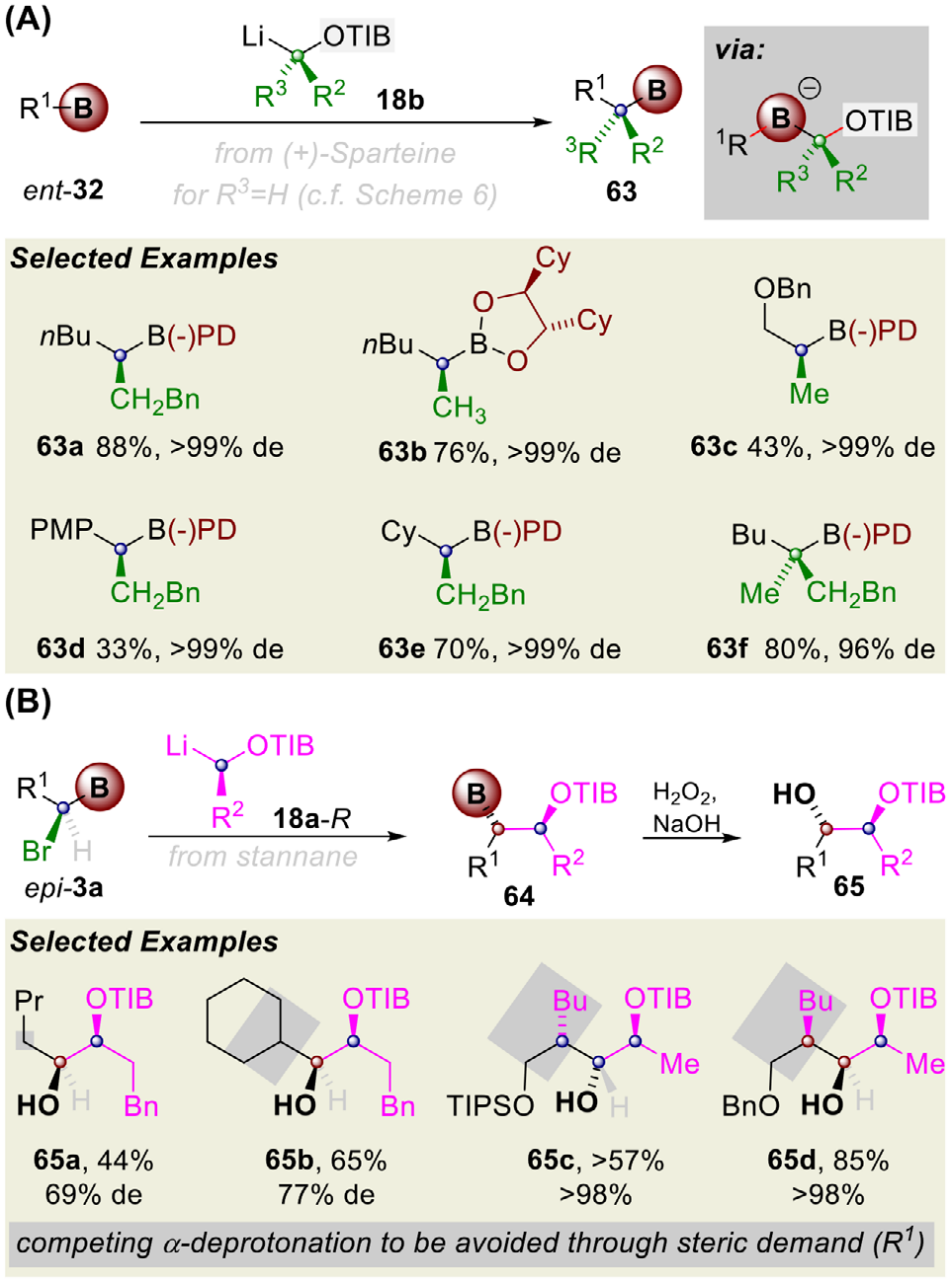
Combining boronic esters and chiral carbenoids.

The Aggarwal group provides excellent procedures for the homologation of pinacol boronates with chiral carbenoids of type **18** [69, 70, 123]. We explored their application to (‐)‐pinanediol boronic esters of type *ent*‐**32** to generate epi‐Matteson products (**63**) [124]. The substrate scope mirrored that of pinacol boronic esters. Aryl, as well as primary and secondary alkyl boronic esters containing pinanediol or DICHED were obtained (**63a**‐**63e**), In general yields were slightly lower than the ones reported for corresponding pinacol boronates. Importantly introduction of disubstituted carbenoids allowed for the generation of tertiary boronic esters (**63f**). Such compounds have only recently become available through MH and the method still suffers from a somewhat limited substrate scope (c.f. Scheme 14). Obviously using both a chiral director and a chiral carbenoid of type **18** only makes sense in an iterative synthesis. There, however, this approach could offer an advantage if a stereocenter with a divergent configuration is required in the middle of an iterative MH sequence. The insertion of lithiated benzylic carbamates into DICHED boronic esters was reported by Becker and Kazmaier, while this review was in production [126].

As depicted in Scheme 17B reaction of carbenoids of type **18a** with α‐halo boronic esters (*epi*‐**3a**) proceeds under substitution of the α‐halogen atom yielding β‐heteroatom substituted boronic esters (**64**). Matteson had previously explored a similar convergent coupling using OMOM‐congeners of type **18** [28]. While preparation of Matteson´s OMOM‐carbenoids was somewhat tedious, Hoppe lithiation provides swift access to the corresponding carbamates and benzoates [127]. However, the resulting substitution products of type **64** are prone to elimination [128, 129]. Best results were obtained with OTIB esters (**18a** *‐R*, generated from stannanes) and concomitant oxidation of **64**. This led to differentially protected glycols of type **65**. Coupling homologation products of primary boronic esters (e.g., *epi*‐**3a** with R^1^ = Bu) was plagued by competing α‐deprotonation (**65a**). This could be mitigated by steric bulk in the β‐position (e.g., the secondary alkyl groups in **65b‐65d**, as highlighted in grey). This way both differentially protected *erythro*‐ and *threo*‐glycols are accessible. Cleavage of the OTIB group can be achieved with LiAlH_4_ to liberate the alcohol [126]. This coupling is particularly suitable for terminating Matteson sequences as shown by the preparation of **65c** and **65d**.

From a retrosynthetic point of view, the reaction shown in Scheme 17B represents the coupling of a chiral a^1^‐reagent (*epi*‐**3a**) with a chiral d^1^‐reagent (**18a**‐*R*). This generates a 1,2‐bifunctional compound. Consequently, a 1,3‐bifunctional disconnection approach can be based on enolates (d^2^) as shown in Scheme 18. Reaction of α‐bromoboronic esters of type **3b** with *trans*‐enolates and subsequent oxidation delivers *anti*‐aldol type products of type **66** [29]. The *n*‐butyl derivative **66a** was prepared with excellent selectivity (97% de), while the isopropyl‐ (**66b**) and phenyl derivatives (**66c**) were only obtained with 86% and 78% de, respectively. This reaction mirrors stereoselective aldol condensations. But in contrast to Evans aldol reactions [8, 9], the absolute configuration is determined by the chiral boronic ester.

**SCHEME 18:**
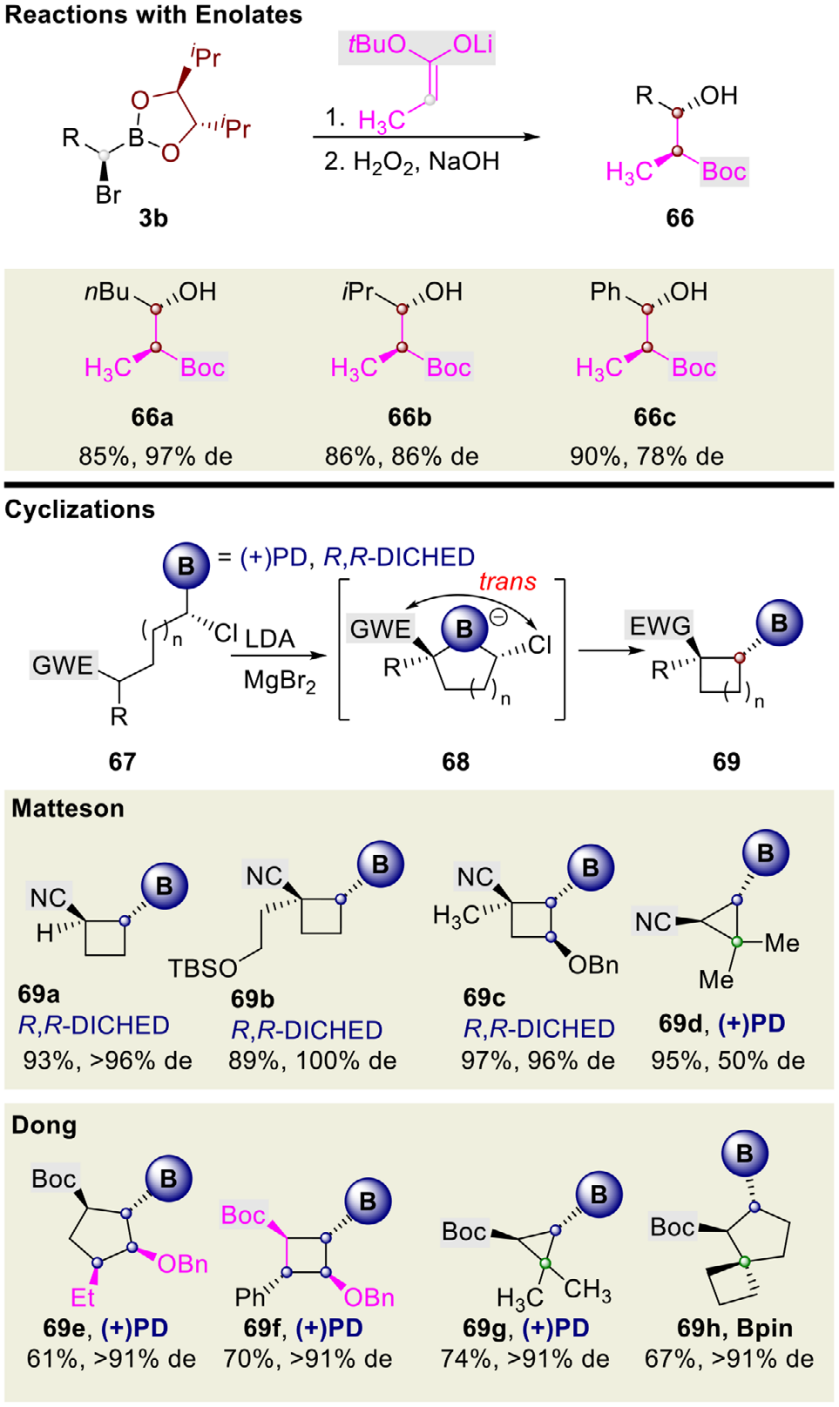
Coupling of MH products with enolates.

As both nitrile groups and *tert*‐butylcarboxylates are tolerated during MHs, substitution with enolates provides opportunities for cyclization reactions. This allows for the synthesis of α‐halo boronic esters of type **67**, which carry an electron withdrawing group (EWG). Its α‐position can be deprotonated with LDA in the presence of the α‐chloro boronic ester. Intramolecular ate complex formation leads to **68** diastereoselectively, in which the electron withdrawing group and the chloride leaving group are in a *trans*‐relationship. MgBr_2_‐mediated 1,2‐boronate rearrangement leads to cycloalkanes of type **69** [130, 131]. Matteson successfully employed nitrile enolates to make cyclobutanes **69a‐c**, which were obtained with excellent diastereoselectivity and yields [129]. His attempt to synthesize cyclopropane **69d** on the other hand delivered a significantly lower diastereomeric excess of 50% [130].

In 2025 Dong and coworkers reported a general strategy for the construction of cyclic motives based on this strategy [132]. They confirmed that the presence of MgBr_2_ is required to obtain good diastereoselectivities, but that the amount can be reduced to 0.4 equivalents. They prepared various ring sizes (3‐ to 6‐membered) from a variety of electron withdrawing groups. To establish a general strategy, they employed β‐borylated *tert*‐butylpropanoate as a key building block. From it a sequence of MHs and subsequent cyclization led to cylcopentane **69e**. The synthesis of cyclobutane **69f** started with MH of PhB(+)PD and introduction of the CH_2_‐Boc group was achieved by use of an enolate derived from *t*‐butylacetate (c.f. Scheme 11). After two more homologations, cyclization led to cyclopropane **69f**. Boc‐cyclopropane **69** **g** was also prepared with a vastly improved diastereoselectivity compared to its nitrile congener **69d** (prepared by Matteson). More complex structures such as spirocycles (**69** **h**) were prepared as well and the method can be considered a general approach to cyclalkanes via iterative MHs.

A key advantage of Matteson reactions is the ability to introduce a variety of masked heteroatom substituents. While the convergent couplings discussed before allow for some flexibility in the synthesis of 1,2‐ and 1,3‐systems, a straight forward *epi*‐Matteson reaction that allows for the introduction of heteroatom substituents would be most desirable. Two strategies to access *epi*‐Matteson products with alkoxy substituents without changing the chiral director have been reported and are depicted in Scheme 19. Carreaux´s approach to the problem was as elegant as it was simple. Why not omit the inversion step of the Matteson substitution, by replacing LiCHCl_2_ with LiCH(OR)_2_? Insertion of LiCH(OEt)_2_ or LiCH(OMe)_2_ into pinanediol boronic esters (**32**) did indeed deliver products such as **70a‐d** in one single step. The lithiated acetals however had to be prepared by tin‐lithium exchange of α‐tributylstannyl acetals [125, 133]. Unfortunately any attempts to insert dibenzyloxy acetals, which would deliver masked hydroxyl groups, failed [132].

**SCHEME 19:**
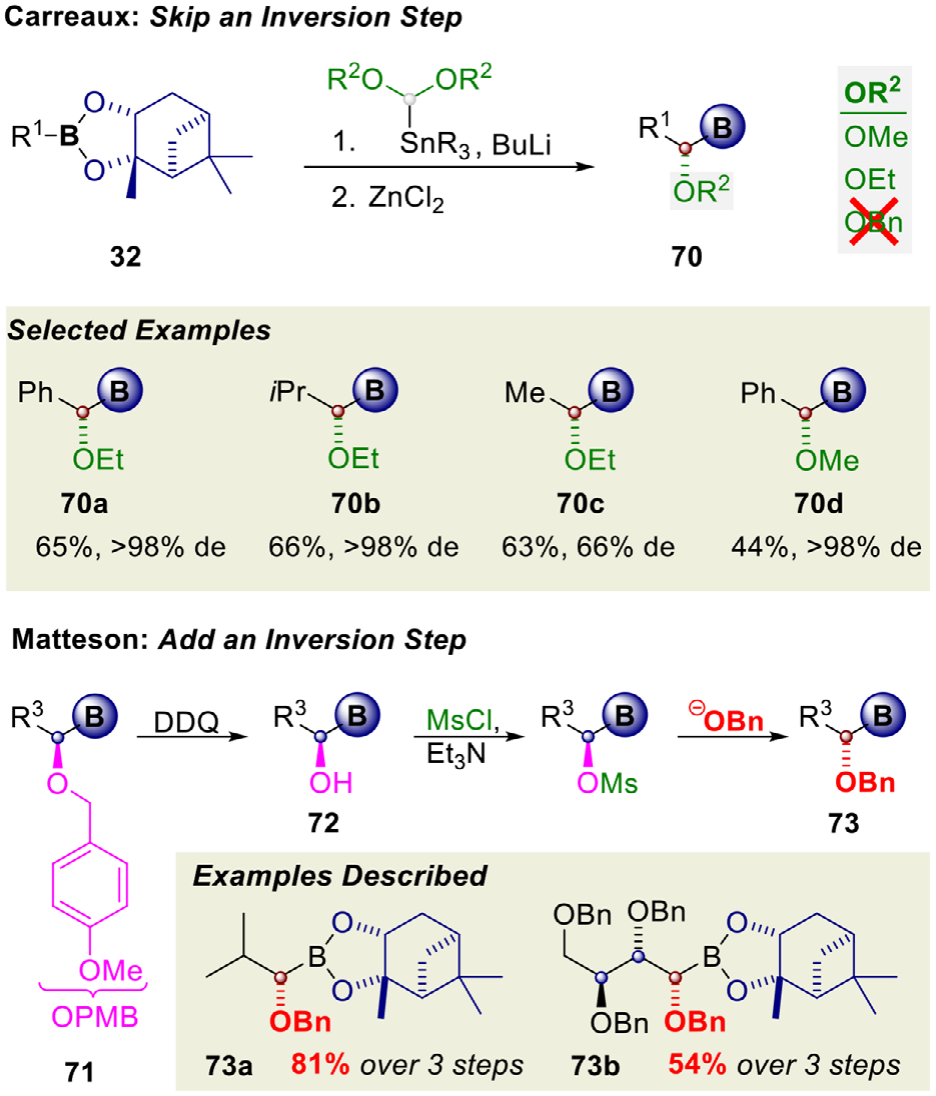
Lithiated acetals used for stereodivergent homologation to α‐alkoxy boronic ester (top); α‐Oxygen deprotection and activation enables SN2 inversion.

Matteson on the other hand approached the problem by adding a third inversion step. α‐PMBO‐boronic esters of type **71** were deprotected with DDQ to yield α‐hydroxyl boronic esters of type **72**. The resulting free alcohol **72** was mesylated and could then be subjected to another substitution with lithium benzyloxide to afford inversion products of type **73** [19]. Although the yields over three consecutive steps were synthetically useful only two examples (**73a** and **73b**) were described and to the best of our knowledge no application of such a sequence has been reported since Matteson´s original publication in 1987. Thus, an easy *epi*‐Matteson reaction that allows for the reliable introduction of heteroatom‐based substituents still remains to be developed.

### 2.4 Comparing Means for Achieving Stereodivergent Outcomes in Substrate Controlled Boronate Homologations

To conclude the second part of this review, an overview of its topics is presented in Scheme 20. In this section we focused on ways of achieving complementary stereochemical outcomes in substrate‐controlled boronate homologations.

**SCHEME 20:**
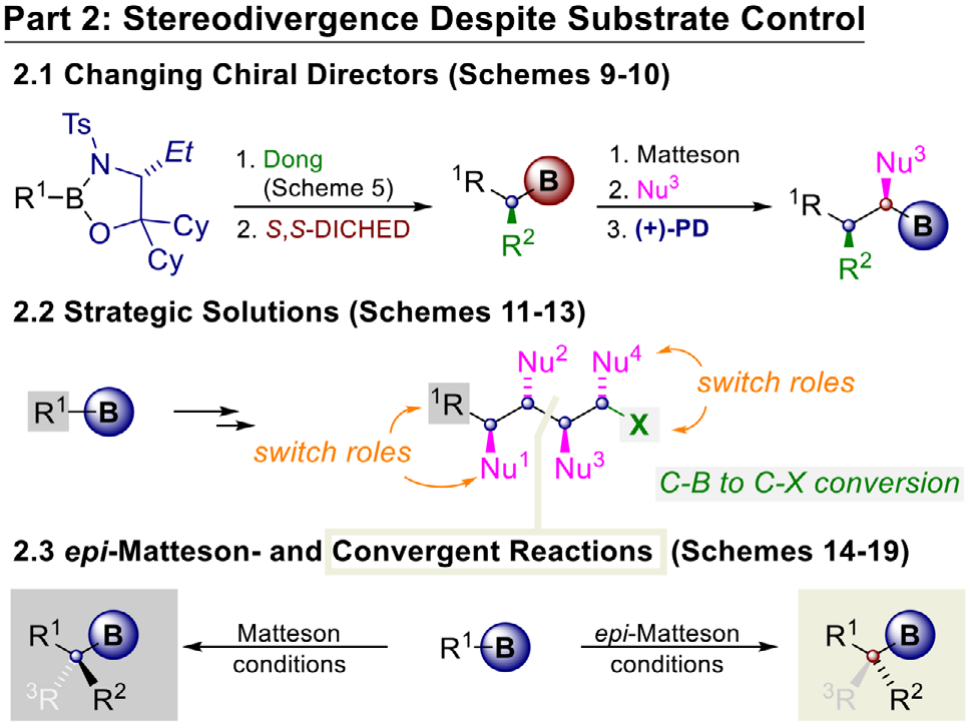
Synopsis of Part 2.

The most straight forward way to achieve stereodivergent outcomes in these reactions is to simply exchange the chiral director (Section 2.1). While overuse of this certainly defeats the efficiency argument we made before, changing the director always remains as a means of last resort. For this it is useful to consider the thermodynamic stability of the chiral directors. Transesterification from DICHED to pinanediol, for example, can be achieved very easily and allows for a simple recovery of DICHED. Some of Dong´s oxazaborolidines required transesterification to pinacol to allow for chromatography. Given this low thermodynamic stability a quick transesterification to DICHED or pinanediol could be convenient. Further stereochemical diversity can be achieved at the beginning and end of a sequence (Section 2.2). By choosing appropriate starting materials or final C‐B conversions, the configuration of both the first and the last stereocenter can be controlled. This control can be expanded by convergent couplings such as the ones described in Section 2.3. There we also discussed several recent developments that allow for achieving stereochemical divergent outcomes upon homologation of DICHED and pinanediol boronic esters. Some of these methods also allow for the synthesis of tertiary boronic esters. Thus, these new *epi*‐Matteson reactions address several disadvantages classically associated with substrate‐controlled boronate homologations.

## 3 Conclusions

Despite the impressive advances in reagent‐controlled boronate homologation chemistry, the classic Matteson reaction remains a highly useful and timely method. Its efficiency is particularly evident upon iterative application, as the chiral director can exert its influence repeatedly. In recent years, several methods have been developed to address the inherent lack of stereochemical variability associated with a single director. Further developments in this area are certainly needed to expand the substrate scope of these *epi*‐Matteson strategies. But even now they already represent a noteworthy expansion of the scope of the venerable Matteson reaction.

## Conflicts of Interest

The author declares no conflict of interest.

## Data Availability

Data sharing is not applicable to this article as no new data were created or analyzed in this study.
